# Seed and soil inoculation with a Bacillus consortium in durum wheat. part II: yield components, grain technological quality and mineral composition

**DOI:** 10.3389/fpls.2026.1920969

**Published:** 2026-08-07

**Authors:** Nicolò Iacuzzi, Noemi Tortorici, Gianluigi Maria Lo Dico, Mauro Sarno, Teresa Tuttolomondo, Antonio Giovino

**Affiliations:** 1 Department of Agricultural, Food and Forest Sciences, University of Palermo, Viale delle Scienze, Palermo, Italy; 2 Istituto Zooprofilattico Sperimentale della Sicilia “A. Mirri”, Palermo, Italy; 3 Council for Agricultural Research and Economics (CREA)—Research Centre for Plant Protection and Certification (CREA-DC), Palermo, Italy

**Keywords:** grain protein concentration, inoculation delivery strategies, Mediterranean agroecosystems, phosphorus-solubilizing bacteria (PSB), plant growth-promoting rhizobacteria (PGPR)

## Abstract

Reducing chemical fertilizers while ensuring food security is a major challenge for Mediterranean cereal systems. This study (Part II) evaluated the effects of a multi-strain *Bacillus* consortium (*B. licheniformis*, *B. methylotrophicus*, *B. megaterium*) on yield components, grain technological quality, and mineral composition of durum wheat (cv. Antalis) under fertilized and unfertilized conditions across multiple delivery strategies (seed, soil, and combined applications) at two contrasting Sicilian sites (Sparacia and Campo Carboj). Results showed that productivity and quality traits were strongly shaped by site-specific factors, with combined seed + soil treatments consistently performing best. At Campo Carboj (less limiting soil), the most intensive combined application closed the yield gap between fertilized and unfertilized plots without compromising technological quality. At Sparacia (heavy clay soil), the consortium strongly stimulated biomass (+141%) and grain protein deposition. Crucially, interaction analysis revealed that the highest protein content (17.85%) occurred under the intensive combined inoculation plus mineral fertilization, indicating synergistic effects with nitrogen inputs. Furthermore, the intensive combined treatment promoted a notable increase in grain phosphorus and induced autonomous calcium biofortification at Campo Carboj, where the unfertilized inoculated plots matched the fertilized ones, offering physiological advantages against terminal stress. A marked accumulation of grain nitrates was also recorded under intensive treatments, providing clear evidence of microbially altered rhizosphere nitrogen dynamics. Overall, the *Bacillus* consortium acted as a complementary bio-stimulatory tool rather than a direct replacement for chemical fertilizers but provided context-dependent compensation and improving nutrient assimilation. Combined multimodal inoculation represents a powerful supplementary strategy to support sustainable durum wheat intensification and biofortification under reduced chemical inputs in Mediterranean field environments.

## 1 Introduction

Durum wheat (*Triticum turgidum L.* subsp. *durum* Desf.) is a cornerstone of the Mediterranean agri-food system and the primary raw material for semolina, pasta, and couscous production. Its economic and nutritional relevance makes both yield stability and grain quality critical targets, particularly in the context of EU sustainability policies that call for a reduction of synthetic fertilizer use by at least 20% and of nutrient losses by at least 50% by 2030 (European Commission, 2020). In this framework, maintaining or improving grain yield and quality while reducing chemical inputs represents one of the central agronomic challenges for cereal production systems.

Grain quality in durum wheat is a multidimensional concept encompassing yield-related traits (grain yield, aboveground biomass, thousand grain weight), technological properties relevant to industrial processing (protein content, dry gluten, yellow index, test weight), and grain mineral composition. These traits are closely interconnected: protein and gluten content determine pasta-making potential through protein polymerisation, which is closely linked to grain nitrogen and sulfur concentrations (Sissons, 2008); test weight and thousand grain weight influence milling efficiency and semolina extraction (Wang and Fu, 2020); and grain mineral accumulation is increasingly relevant for nutritional quality and as an indicator of efficient source-to-sink nutrient translocation. While the benefits of plant growth-promoting rhizobacteria (PGPR) on cereal vegetative biomass are well-established, their capacity to modulate grain technological properties and mineral profiles under restricted fertilization remains an emerging field of research. Recent studies have shown that specific *Bacillus* strains can enhance nitrogen and micronutrient translocation towards the developing grain, thereby affecting protein composition and rheological traits (Ait-El-Mokhtar et al., 2023; Saeed et al., 2021). However, field responses are often highly variable and strongly dependent on the specific bacterial-genotype-environment interactions (Mącik et al., 2020). In Mediterranean environments, all of these traits are sensitive to the interaction between soil nutrient availability, water status, and terminal thermal stress during grain filling, making their simultaneous improvement challenging under reduced fertilization.

Phosphate-solubilizing bacteria (PSB) and plant growth-promoting rhizobacteria (PGPR), particularly within the genus *Bacillus*, have been proposed as biostimulant tools capable of improving nutrient acquisition and plant physiology without the environmental costs of mineral fertilization (Etesami and Adl, 2020; Pang et al., 2024). The mechanisms by which these microorganisms may benefit crop performance include phosphorus mobilization from sparingly soluble soil fractions, production of phytohormones (auxins and cytokinins) that stimulate root development, synthesis of ACC deaminase that reduces ethylene-induced stress inhibition, and modulation of rhizosphere biochemistry that enhances the overall nutritional status of the plant (Blake et al., 2021; Kudoyarova et al., 2019). Under Mediterranean field conditions, where P immobilization in alkaline and calcareous soils frequently restricts crop performance, these mechanisms are particularly relevant.

The companion paper to the present study (Iacuzzi et al., 2026, Part I) evaluated the effects of a multi-strain *Bacillus* consortium (*B. licheniformis, B. methylotrophicus, B. megaterium*) on soil available phosphorus (P_2_O_5_) and on plant morphological and physiological indicators across the 2023–2024 growing season at two contrasting Sicilian sites (Sparacia and Campo Carboj), under fertilized and unfertilized conditions and across multiple delivery strategies (seed coating, soil spraying at three rates, and combined applications) aimed at optimizing bacterial persistence and root colonization across different crop phenological stages. The key findings of Part I demonstrated that microbial inoculation significantly increased soil P_2_O_5_ availability, especially in the more P-constraining clay-rich soil at Sparacia, and improved canopy-level physiological indicators including relative leaf chlorophyll content measured via SPAD meter, stomatal conductance, and Normalized Difference Vegetation Index (NDVI), particularly in the absence of mineral fertilization. These results suggest that the consortium partially compensated for fertilizer withdrawal by improving soil P dynamics and maintaining crop physiological functioning during the vegetative and reproductive stages.

However, improvements in soil nutrient availability and canopy physiological status do not necessarily translate into measurable gains in final grain yield or technological quality. The relationship between vegetative performance and productive output is strictly governed by source–sink dynamics during grain filling, which in Mediterranean environments are frequently disrupted by terminal water and heat stress. Furthermore, even when yield increases are recorded, they may be associated with dilution effects on grain protein concentration-a well-known trade-off in cereal agronomy-which would be detrimental to industrial quality. urthermore, even when yield increases are recorded, they may be associated with dilution effects on grain protein concentration—a well-known trade-off in cereal agronomy (Kibite and Evans, 1984; Simmonds, 1995)—which would be detrimental to industrial quality. In recent years, a major challenge in biostimulant research has been identifying microbial formulations capable of breaking this negative correlation by simultaneously upgrading both yield components and grain protein deposition under nitrogen-limiting conditions (Popko et al., 2018). The extent to which microbial inoculation can simultaneously support yield and preserve or improve quality under reduced fertilization therefore remains an open question with high practical relevance.

The present study (Part II) addresses this gap by analysing the yield components, grain technological quality, and chemical and mineral composition of durum wheat (cv. Antalis, a modern high-yielding elite cultivar widely grown in the Mediterranean basin and characterized by high industrial quality potential but high nitrogen requirements) in response to the same *Bacillus* consortium tested in Part I, across the same two Sicilian sites and under the same range of delivery strategies and fertilization regimes. The study was guided by three main hypotheses: (i) that microbial inoculation would improve grain yield and aboveground biomass through enhanced nutrient acquisition, with a particularly pronounced response expected in the more nutrient-limited environment of Sparacia, and a potential reduction of the yield gap between fertilized and unfertilized treatments in the more productive environment of Campo Carboj; (ii) that the most intensive combined applications (S6, S7) would produce the most robust improvements in technological quality traits, particularly protein content and test weight, without a yield–quality trade-off, by ensuring prolonged bacterial persistence in the rhizosphere during the critical post-anthesis grain filling phase (Mamun et al., 2024); and (iii) that grain mineral accumulation, particularly phosphorus, would reflect the improvements in soil P availability documented in Part I, providing a mechanistic link between soil processes and grain nutritional quality through microbial-driven biofortification (Silva et al., 2023). Together, Parts I and II are designed to provide a comprehensive assessment of the agronomic and qualitative value of *Bacillus*-based inoculation as a component of more sustainable fertilization programs for Mediterranean durum wheat.

## 2 Materials and methods

### 2.1 Experimental sites, plant material, and design

The field experiment was conducted during the 2023–2024 winter–spring growing season at two experimental farms in Sicily (Italy): “Campo Carboj” (Castelvetrano, TP; 37° 51’ 17″ N, 12° 53’ 40″ E; 30 m a.s.l.) and “Sparacia” (Cammarata, AG; 37° 38’ 08.8″ N, 13° 45’ 46.4″ E; 390 m a.s.l.). Both sites represent contrasting pedoclimatic conditions: Campo Carboj has a sandy-loam soil (59% sand, 28% clay, pH 8.14) with relatively high baseline fertility, while Sparacia is characterized by a heavy clay soil (62% clay, 5% sand, pH 7.89) prone to strong phosphorus immobilization. Full details of the soil physico-chemical properties, crop management, microbial inoculant (Glysssp^®^; Hello Nature, Italy; *B. licheniformis*, *B. methylotrophicus*, *B. megaterium*, each at 1.0 × 10^8^ CFU g^-1^), application treatments (S0–S7), fertilization regimes (fertilized, F; unfertilized, noF), and randomized complete block design (three replicates, 9 m^2^ plots) are reported in the companion paper (Iacuzzi et al., 2026, Part I). The three *Bacillus* species (*B*. *licheniformis*, *B*. *methylotrophicus*, and *B*. *megaterium*) constituting the commercial consortium were selected and co-formulated by the manufacturer based on demonstrated inter-strain biochemical compatibility. Specifically, the industrial formulation process screens for the absence of mutual antibiosis or bacteriocin-mediated inhibition among the strains, ensuring synergistic metabolic co-existence during both storage and rhizosphere establishment. Sowing took place on 11 December 2023 at both sites. Harvest was performed on 15 June 2024.

### 2.2 Grain and biomass yield

At harvest, grain yield and aboveground biomass yield were determined for each experimental plot and converted to q ha^-^¹. Grain yield was measured after threshing and cleaning of the harvested material, whereas aboveground biomass yield was determined from the total harvested aerial plant material. The weight of 1000 seeds (TKW) [g] was calculated from the average weight of five sets of 100 seeds per replication.

### 2.3 Grain technological quality

For each replication, three 500.0 g seed samples were analyzed using the Infratec NOVA instrument (Foss Analytics, Italy), which utilizes near-infrared (NIR) transmittance technology, to determine the following qualitative, and commercial parameters: dry gluten (DG) [%], yellow index (YI) [%], test weight (TW) [kg hL^-^¹] and protein content [%].

### 2.4 Grain chemical and mineral composition

Grain chemical and mineral composition was determined on homogenized grain samples. Nitrate, phosphate, calcium, and ash content were analysed according to laboratory procedures provided by the Istituto Zooprofilattico Sperimentale della Sicilia. Nitrate concentration was determined by ion chromatography. Approximately 1 g of homogenized sample was weighed into a 100 mL volumetric flask, mixed with approximately 70 mL of deionized water, and extracted in a water bath at 50 ± 1 °C for 30 min with occasional shaking. After cooling to room temperature, the extract was quantitatively filtered into a 100 mL volumetric flask. The residue and filter were washed with deionized water, and the final volume was adjusted to 100 mL. The solution was then filtered through 0.45 µm syringe filters and transferred into chromatographic vials. Nitrate was quantified using an ICS 5000 ion chromatograph (Thermo Fisher Scientific) equipped with a conductivity detector, a Rheodyne injector with a 50 µL loop, a DRS 600 suppressor, an IonPac AS9-HC analytical column (4 × 250 mm), and an IonPac AG9-HC guard column (4 × 50 mm). The chromatographic separation was performed under isocratic conditions using 9 mM sodium carbonate as eluent, at a flow rate of 1.0 mL min^-^¹ and an injection volume of 50 µL. Quantification was performed by external calibration using nitrate standard solutions. Phosphate was determined by ion chromatography after dry ashing and alkaline extraction. Approximately 5 g of sample were incinerated at 550 °C to constant weight. The ash residue was transferred into a 100 mL volumetric flask, extracted with approximately 30 mL of 0.1 M NaOH under boiling conditions with occasional shaking, cooled to room temperature, quantitatively filtered, and brought to volume with deionized water. The extract was filtered through 0.45 µm syringe filters before chromatographic analysis. Phosphate was quantified using the same ion chromatographic system described above, with external calibration based on phosphate standard solutions. Calcium concentration was determined by ion chromatography after acid extraction of the ash residue. Approximately 5 g of ashed sample were treated with 30 mL of 0.5 M methanesulfonic acid, brought to a final volume of 100 mL with deionized water, filtered through 0.45 µm filters, and injected into the chromatographic system. Calcium was analysed using a Dionex ICS 5000 ion chromatograph equipped with an ASRS-300 suppressor, an IonPac CS12 analytical column (4 × 250 mm), an IonPac CG12A guard column (4 × 50 mm), and Chromeleon software. Ash content was determined gravimetrically. Approximately 5 g of sample were weighed into crucibles, pre-charred over a Bunsen flame, and then incinerated in a muffle furnace at 550 °C until constant weight. Ash content was calculated as the ratio between the mass of the residue after incineration and the initial sample mass and expressed as percentage.

### 2.5 Statistical analysis

All analyses were carried out using Minitab^®^ Statistical Software (version 21.3; Minitab LLC, State College, PA, USA). Data were subjected to analysis of variance (ANOVA) using a randomized complete block design. Normality and homogeneity of variance were verified prior to analysis. When significant effects were detected (p ≤ 0.05), mean separation was performed using Tukey’s Honest Significant Difference (HSD) *post-hoc* test, following the methodology described by Gomez and Gomez (1984).

## 3 Results

### 3.1 Productivity traits and yield response

Analysis of variance showed that both microbial application (A) and mineral fertilization (F) significantly affected grain yield, aboveground biomass yield, and thousand grain weight (TGW), although the magnitude of the response varied according to the experimental site and the measured trait (**Table 1**). For grain yield, the effect of microbial application was particularly pronounced at Sparacia, whereas fertilization had a stronger effect at Carboj. The A × F interaction was significant for grain yield only at Carboj, while it was not significant at Sparacia. For biomass yield, all model terms were significant at both sites, with the strongest responses observed at Sparacia. TGW was significantly affected by both application and fertilization in both environments, and the A × F interaction was also significant, especially at Carboj.

**Table 1:** Analysis of variance (ANOVA) results for grain yield (q ha^-^¹), biomass yield (q ha^-^¹) and thousand grain weight (g) in response to application (A), fertilization (F) and their interaction (A × F) at the two experimental sites (Sparacia and Carboj).

Source of variation		df	Yield [q ha^-1^]	Biomass yield [q ha^-1^]	Thousand grain weight [g]
Application (A)	Sparacia	7	391.12 ***	177.20 ***	5.37 ***
	Carboj		54.66 **	17.16 ***	13.34 ***
Fertilization (F)	Sparacia	1	57.87 ***	671.15 ***	120.01 ***
	Carboj		669.18 ***	14.30 ***	219.49 ***
A × F	Sparacia	7	0.96 ns	32.76 **	2.82 *
	Carboj		22.42 *	5.11 ***	8.13 ***

Values are Fisher’s F statistics; degrees of freedom (df) are reported. Significance levels: ***p ≤ 0.001, **p ≤ 0.01, *p ≤ 0.05; ns, not significant.

Considering the main effect of microbial application, grain yield increased progressively with increasing treatment intensity at both sites (**Table 2**). At Sparacia, grain yield rose from 15.11 q ha^-1^ in the untreated control (S0) to 20.09 q ha^-1^ in S7, corresponding to an increase of approximately 33%. Treatments S4, S5, S6, and S7 formed the highest statistical group, indicating that the most intensive microbial applications produced comparable yield responses. At Carboj, the response was even more pronounced in absolute terms, with grain yield increasing from 21.71 q ha^-1^ in S0 to 29.33 q ha^-1^ in S7, corresponding to a 35.1% increase. In this site, S7 was the most productive treatment and was significantly higher than the other application levels.

**Table 2:** Effects of application and fertilization on grain yield, biomass yield and thousand grain weight at the two experimental sites (Sparacia and Carboj).

Treatments	Yield [q ha^-1^]		Biomass yield [q ha^-1^]		Thousand grain weight [g]	
	Sparacia	Carboj	Sparacia	Carboj	Sparacia	Carboj
Application (A)						
S0	15.11 ± 0.54 c	21.71 ± 2.16 f	32.13 ± 2.46 f	75.62 ± 3.49 e	35.65 ± 1.40 bc	40.84 ± 0.54 d
S1	17.45 ± 0.81 b	22.55 ± 1.09 ef	60.55 ± 9.40 c	82.14 ± 0.94 bcd	35.30 ± 1.26 c	44.66 ± 1.13 bc
S2	17.35 ± 0.75 b	23.34 ± 2.32 de	39.44 ± 3.20 e	81.49 ± 2.74 cde	36.41 ± 1.29 abc	43.74 ± 1.35 cd
S3	17.88 ± 0.65 b	24.57 ± 1.28 cd	54.04 ± 8.49 d	80.14 ± 1.64 de	36.55 ± 0.88 abc	46.06 ± 1.86 bc
S4	19.40 ± 0.69 a	24.69 ± 1.23 cd	61.84 ± 8.06 c	89.13 ± 0.84 a	37.60 ± 0.51 ab	45.95 ± 2.34 bc
S5	19.23 ± 0.71 a	25.48 ± 1.56 bc	71.29 ± 5.63 b	87.48 ± 1.11 ab	35.87 ± 0.82 bc	46.03 ± 2.37 bc
S6	19.75 ± 0.84 a	26.31 ± 0.98 b	75.53 ± 3.03 ab	86.04 ± 1.32 abc	37.76 ± 0.62 ab	47.65 ± 2.48 ab
S7	20.09 ± 0.65 a	29.33 ± 0.39 a	77.41 ± 1.84 a	91.87 ± 0.48 a	38.56 ± 1.04 a	50.17 ± 3.26 a
p	0.000	0.000	0.000	0.000	0.000	0.000
Fertilization (F)						
no Fertilization (no F)	16.76 ± 0.31 b	21.79 ± 0.74 b	47.69 ± 3.45 b	82.52 ± 1.63 b	34.79 ± 0.41 b	41.71 ± 0.38 b
Fertilization (F)	19.81 ± 0.36 a	27.70 ± 0.30 a	70.37 ± 3.63 a	85.96 ± 0.85 a	38.63 ± 0.26 a	49.57 ± 0.98 a
p	0.000	0.000	0.000	0.001	0.000	0.000

Means and standard errors are reported. Within each column, values with different letters are significantly different at p ≤ 0.05, according to Tukey’s HSD test. S0: soil spraying with water only; S1: seed coating at a dose of 100 g q^-^¹ of seeds; S2: soil spraying after sowing at a dose of 1 kg ha^-^¹ of microbial complex; S3: soil spraying after sowing at a dose of 2 kg ha^-^¹ of microbial complex; S4: soil spraying after sowing at a dose of 3 kg ha^-^¹ of microbial complex; S5: S1 + S2; S6: S1 + S3; S7: S1 + S4.

A similar pattern was observed for biomass yield, although the magnitude of the response differed strongly between the two sites. At Sparacia, biomass increased from 32.13 q ha^-1^ in S0 to 77.41 q ha^-1^ in S7, corresponding to an increase of approximately 141%. This indicates a strong stimulation of vegetative growth following microbial application, particularly under the combined seed + soil treatments. At Carboj, biomass values were generally higher than at Sparacia, but the relative increase was smaller, ranging from 75.62 q ha^-1^ in S0 to 91.87 q ha^-1^ in S7. For TGW, the effect of microbial application was moderate at Sparacia, increasing from 35.65 g in S0 to 38.56 g in S7, whereas a clearer response was observed at Carboj, where TGW increased from 40.84 g in S0 to 50.17 g in S7.

Mineral fertilization significantly increased all productivity traits at both sites (**Table 2**). Grain yield increased by 18.2% at Sparacia and by 27.1% at Carboj compared with the unfertilized treatment. Biomass yield also increased under fertilization, although the response was much stronger at Sparacia (+47.6%) than at Carboj (+4.2%). TGW increased by 11.0% at Sparacia and by 18.8% at Carboj, confirming the positive effect of mineral fertilization on grain filling.

**Figure 1:**
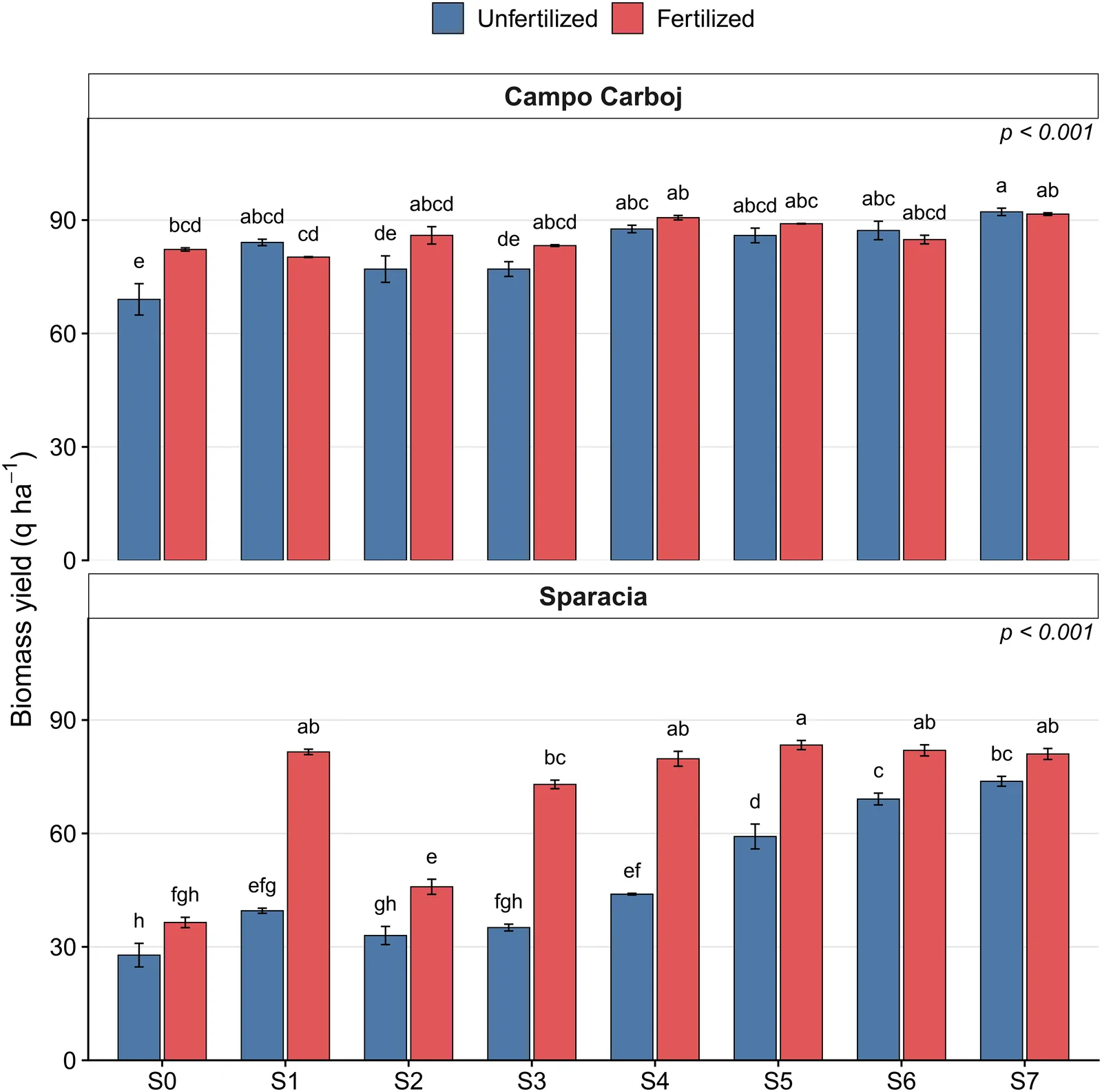
Influence of the application × fertilization interaction on aboveground biomass yield at the two sicilian sites. Campo Carboj site; Sparacia site. For each data series, values with different letters are significantly different at p ≤ 0.05, according to Tukey’s HSD test. Error bars represent standard error. S0: soil spraying with water only; S1: seed coating at a dose of 100 g q-1 of seeds; S2: soil spraying after sowing at a dose of 1 kg ha-1 of microbial complex; S3: soil spraying after sowing at a dose of 2 kg ha-1 of microbial complex; S4: soil spraying after sowing at a dose of 3 kg ha-1 of microbial complex; S5: S1 + S2; S6: S1 + S3; S7: S1 + S4; F, fertilization; noF, unfertilized.

The A × F interaction provided additional information on the site-specific response to the combined treatments. For biomass yield, the interaction was significant at both sites (**Figure 1**). At Carboj, biomass ranged from 69.02 q ha^-1^ in S0 × noF to 92.17 q ha^-1^ in S7 × noF. Interestingly, S7 × noF did not differ significantly from S7 × F, suggesting that, under the conditions of this site, the highest microbial application partly reduced the biomass gap between fertilized and unfertilized plots. At Sparacia, the interaction was more marked, with values ranging from 27.81 q ha^-1^ in S0 × noF to 83.38 q ha^-1^ in S5 × F. In this environment, fertilization strongly enhanced biomass production, particularly when combined with microbial application.

**Figure 2:**
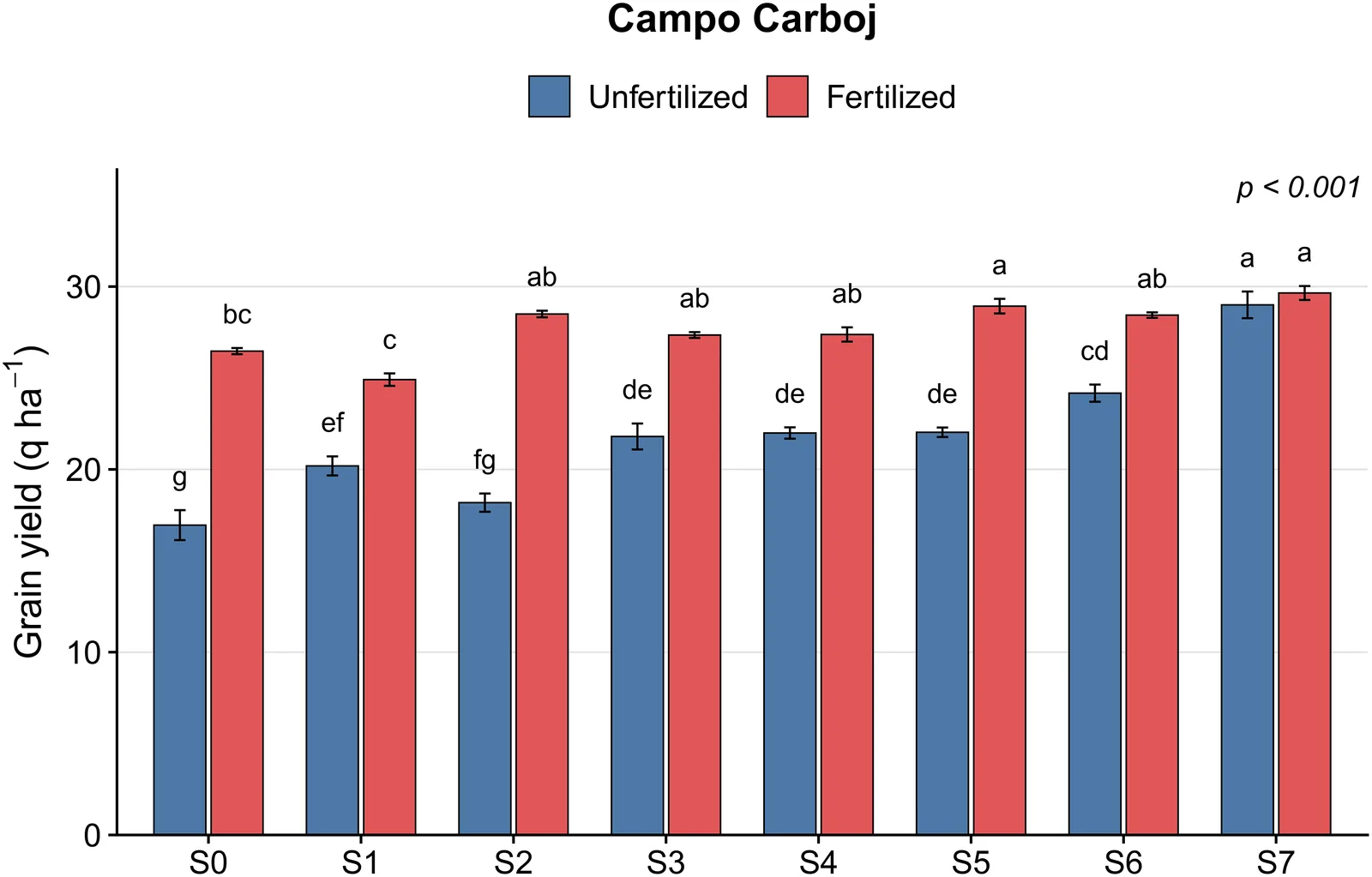
Influence of the application × fertilization interaction on grain yield at Campo Carboj. For each data series, values with different letters are significantly different at p ≤ 0.05, according to Tukey’s HSD test. Error bars represent standard error. S0: soil spraying with water only; S1: seed coating at a dose of 100 g q-1 of seeds; S2: soil spraying after sowing at a dose of 1 kg ha-1 of microbial complex; S3: soil spraying after sowing at a dose of 2 kg ha-1 of microbial complex; S4: soil spraying after sowing at a dose of 3 kg ha-1 of microbial complex; S5: S1 + S2; S6: S1 + S3; S7: S1 + S4; F, fertilization; noF, unfertilized.

For grain yield, the A × F interaction was significant only at Carboj (**Figure 2**). Yield ranged from 16.95 q ha^-^¹ in S0 × noF to 29.65 q ha^-1^ in S7 × F. The highest-yielding group included S7 × F, S7 × noF, and S5 × F, which did not differ significantly from each other. This pattern indicates that the effect of fertilization depended on the level of microbial application: the yield advantage of fertilization was greatest in the control and low-intensity treatments, whereas it became minimal in S7, where fertilized and unfertilized plots produced statistically comparable yields.

**Figure 3:**
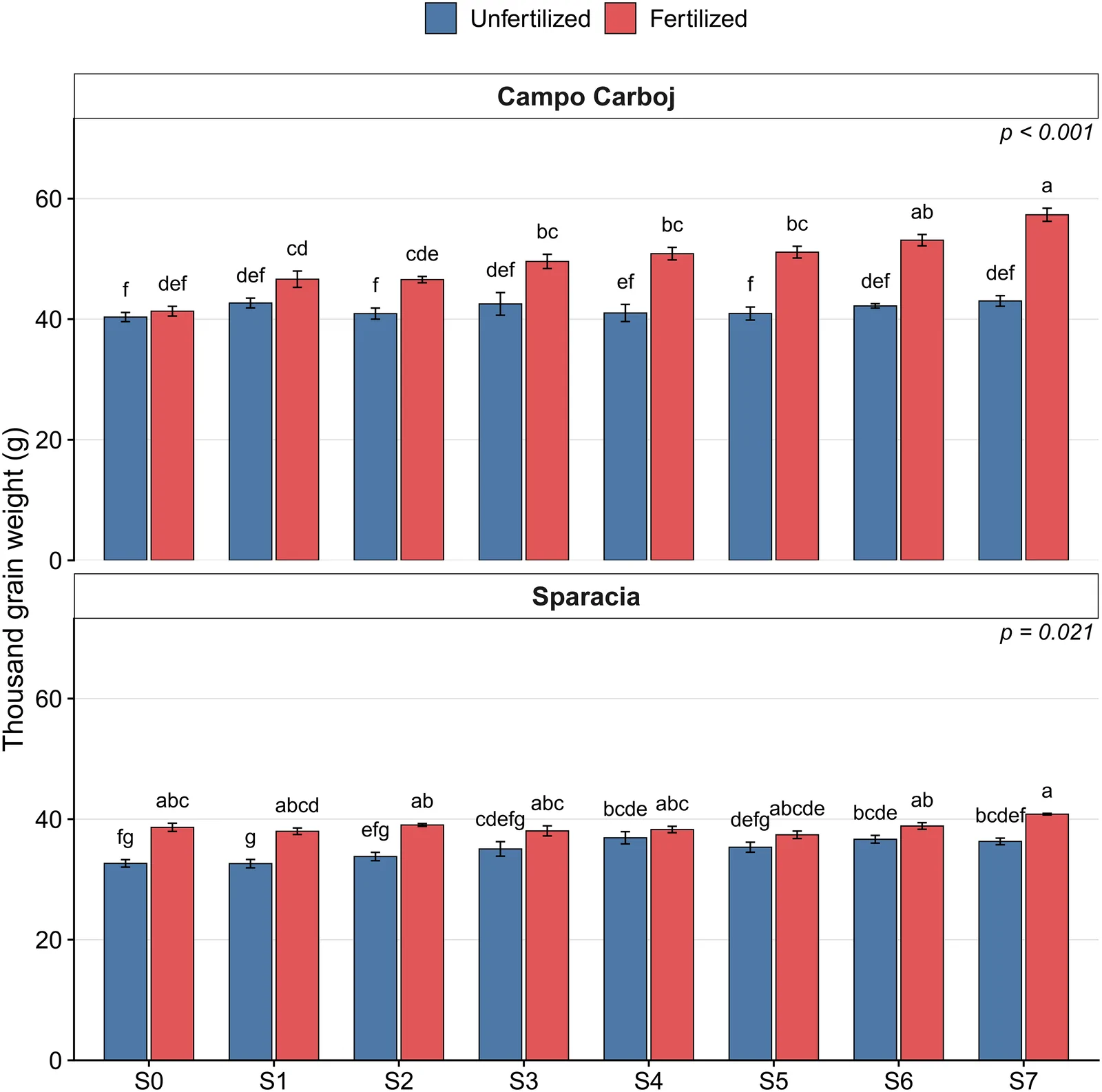
Influence of the application × fertilization interaction on thousand grain weight at the two sicilian sites. Campo Carboj site; Sparacia site. For each data series, values with different letters are significantly different at p ≤ 0.05, according to Tukey’s HSD test. Error bars represent standard error. S0: soil spraying with water only; S1: seed coating at a dose of 100 g q-1 of seeds; S2: soil spraying after sowing at a dose of 1 kg ha^-^¹ of microbial complex; S3: soil spraying after sowing at a dose of 2 kg ha-1 of microbial complex; S4: soil spraying after sowing at a dose of 3 kg ha-1 of microbial complex; S5: S1 + S2; S6: S1 + S3; S7: S1 + S4; F, fertilization; noF, unfertilized.

The interaction for TGW showed a similar pattern (**Figure 3**). At Carboj, TGW ranged from 40.35 g in S0 × noF to 57.32 g in S7 × F, with S7 × F and S6 × F forming the highest statistical group. At Sparacia, TGW ranged from 32.62 g in S1 × noF to 40.82 g in S7 × F. In this site, the differences among fertilized treatments were less pronounced, but fertilized plots generally showed higher TGW values than their corresponding unfertilized treatments.

### 3.2 Technological quality

The response of technological quality traits was strongly dependent on both site and parameter (**Table 3**). Dry gluten was significantly affected by microbial application only at Sparacia, whereas mineral fertilization significantly increased dry gluten at both sites. The A × F interaction was not significant for dry gluten in either environment, indicating that the effects of microbial application and fertilization were mainly additive or independent for this trait.

**Table 3:** Analysis of variance (ANOVA) results for dry gluten (%), yellow index (%), test weight (kg hL^-^¹) and protein (%) in response to application (A), fertilization (F) and their interaction (A × F) at the two experimental sites (Sparacia and Carboj).

Source of variation		df	Dry gluten [%]	Yellow index [%]	Test weight [kg hL^−1^]	Protein [%]
Application (A)	Sparacia	7	3.97 **	3.57 **	18.61 ***	18.15 ***
	Carboj		0.60 ns	1.37 ns	2.67 *	31.95 ***
Fertilization (F)	Sparacia	1	109.31 ***	0.45 ns	435.89 ***	41.15 ***
	Carboj		572.76 ***	138.28 ***	339.17 ***	0.53 ns
A × F	Sparacia	7	1.42 ns	3.68 **	23.65 ***	2.86 *
	Carboj		0.10 ns	1.05 ns	0.70 ns	0.85 ns

Values are Fisher’s F statistics; degrees of freedom (df) are reported. Significance levels: ***p ≤ 0.001, **p ≤ 0.01, *p ≤ 0.05; ns = not significant.

At Sparacia, dry gluten increased from 10.57% in S0 to 11.92% in S7, with S7 among the highest-performing treatments (**Table 4**). At Carboj, microbial application did not significantly affect dry gluten, and all application levels belonged to the same statistical group. Fertilization had a much stronger effect: dry gluten increased from 10.41% to 12.18% at Sparacia and from 8.86% to 13.24% at Carboj.

**Table 4:** Effects of application and fertilization on grain technological quality: dry gluten (%), yellow index (%), test weight (kg hL^-^¹) and protein (%) at the two experimental sites (Sparacia and Carboj).

Treatments	Dry gluten [%]		Yellow index [%]		Test weight [kg hL^−1^]		Protein [%]	
	Sparacia	Carboj	Sparacia	Carboj	Sparacia	Carboj	Sparacia	Carboj
Application (A)								
S0	10.57 ± 0.47 c	10.79 ± 0.99 a	23.46 ± 0.66 b	23.95 ± 0.97 a	71.96 ± 0.97 c	76.06 ± 1.87 b	10.94 ± 0.26 c	10.38 ± 0.08 e
S1	11.53 ± 0.35 ab	11.16 ± 1.02 a	23.93 ± 0.65 ab	24.70 ± 0.65 a	72.90 ± 0.59 c	77.86 ± 1.78 ab	13.38 ± 0.36 b	12.19 ± 0.41 c
S2	10.68 ± 0.53 bc	11.05 ± 1.04 a	23.29 ± 0.87	24.80 ± 0.86 a	74.40 ± 2.02 b	76.83 ± 1.87 ab	11.12 ± 0.54 c	11.05 ± 0.11 cde
S3	11.28 ± 0.43 abc	11.02 ± 1.01 a	24.15 ± 0.25 ab	24.80 ± 0.53 a	74.80 ± 1.74 ab	77.73 ± 2.04 ab	12.24 ± 0.49 bc	10.98 ± 0.33 de
S4	11.49 ± 0.56 ab	11.09 ± 1.05 a	23.84 ± 0.41 b	25.29 ± 0.70 a	75.15 ± 1.29 ab	77.45 ± 2.01 ab	12.18 ± 0.53 bc	11.69 ± 0.23 cd
S5	11.72 ± 0.46 ab	10.98 ± 1.0 a	23.56 ± 0.66 b	24.46 ± 0.68 a	75.21 ± 1.02 ab	77.45 ± 1.88 ab	12.45 ± 0.99 bc	12.24 ± 0.25 c
S6	11.16 ± 0.31 abc	10.89 ± 1.02 a	23.48 ± 0.50 b	24.56 ± 0.70 a	75.03 ± 0.73 ab	77.56 ± 1.58 ab	13.35 ± 0.96 b	13.62 ± 0.28 b
S7	11.92 ± 0.52 a	11.46 ± 0.91 a	25.81 ± 0.56 a	25.04 ± 0.52 a	75.83 ± 0.28 a	79.50 ± 1.54 a	16.61 ± 0.56 a	14.85 ± 0.15 a
p	0.003	0.752	0.006	0.253	0.000	0.027	0.000	0.000
Fertilization (F)								
no Fertilization (no F)	10.41 ± 0.14 b	8.86 ± 0.13 b	23.84 ± 0.20 a	23.28 ± 0.18 b	72.17 ± 0.41 b	73.67 ± 0.31 b	11.83 ± 0.38 b	12.06 ± 0.30 a
Fertilization (F)	12.18 ± 0.15 a	13.24 ± 0.09 a	24.04 ± 0.34 a	26.12 ± 0.16 a	76.65 ± 0.39 a	81.44 ± 0.34 a	13.73 ± 0.44 a	12.19 ± 0.32 a
p	0.000	0.000	0.509	0.000	0.000	0.000	0.000	0.473

Means and standard errors are reported. Within each column, values with different letters are significantly different at p ≤ 0.05, according to Tukey’s HSD test. S0: soil spraying with water only; S1: seed coating at a dose of 100 g q^-^¹ of seeds; S2: soil spraying after sowing at a dose of 1 kg ha^-^¹ of microbial complex; S3: soil spraying after sowing at a dose of 2 kg ha^-^¹ of microbial complex; S4: soil spraying after sowing at a dose of 3 kg ha^-^¹ of microbial complex; S5: S1 + S2; S6: S1 + S3; S7: S1 + S4.

**Figure 4:**
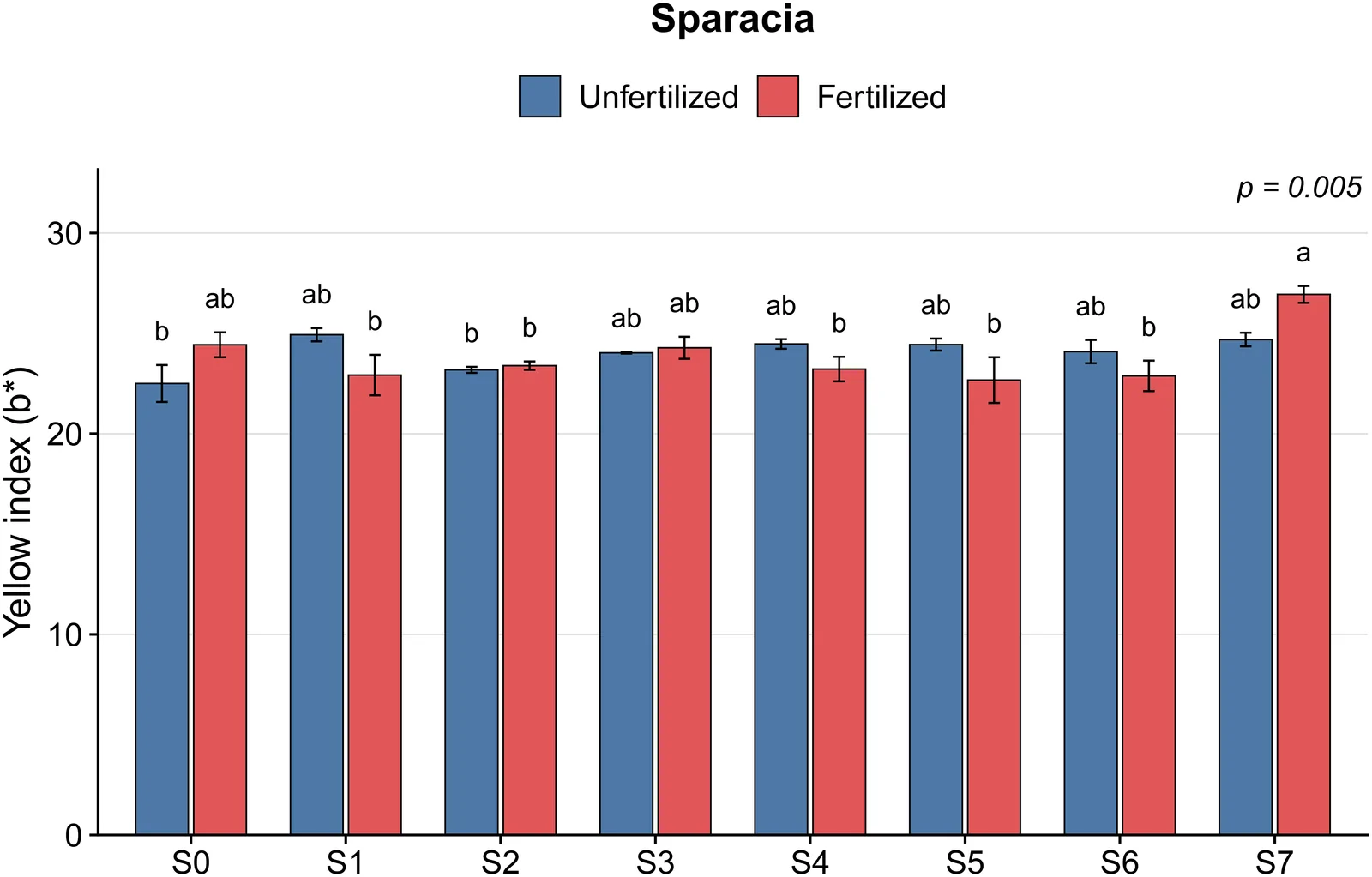
Influence of the application × fertilization interaction on yellow index at Sparacia. For each data series, values with different letters are significantly different at p ≤ 0.05, according to Tukey’s HSD test. Error bars represent standard error. S0: soil spraying with water only; S1: seed coating at a dose of 100 g q-1 of seeds; S2: soil spraying after sowing at a dose of 1 kg ha-1 of microbial complex; S3: soil spraying after sowing at a dose of 2 kg ha-1 of microbial complex; S4: soil spraying after sowing at a dose of 3 kg ha-1 of microbial complex; S5: S1 + S2; S6: S1 + S3; S7: S1 + S4; F, fertilization; noF, unfertilized.

The yellow index showed a different response pattern. At Sparacia, it was significantly affected by microbial application and by the A × F interaction, whereas fertilization alone was not significant. At Carboj, the opposite pattern was observed: fertilization significantly increased the yellow index, while microbial application and the A × F interaction were not significant. At Sparacia, the interaction analysis showed values ranging from 22.50% to 26.94%, with S7 × F reaching the highest value (**Figure 4**).

Test weight was significantly affected by microbial application and fertilization at both sites, while the A × F interaction was significant only at Sparacia (**Table 3**). At Sparacia, test weight increased from 71.96 kg hL^-^¹ in S0 to 75.83 kg hL^-1^ in S7. At Carboj, values ranged from 76.06 kg hL^-1^ in S0 to 79.50 kg hL^-1^ in S7. Fertilization caused a clear increase in test weight at both sites, from 72.17 to 76.65 kg hL^-^¹ at Sparacia and from 73.67 to 81.44 kg hL^-1^ at Carboj.

Protein content was significantly affected by microbial application at both sites, while fertilization and the A × F interaction were significant only at Sparacia (**Table 3**). At Sparacia, protein content increased from 10.94% in S0 to 16.61% in S7. Notably, the increase between S6 (13.35%) and S7 (16.61%) was particularly marked relative to the stepwise progression observed in other treatments, suggesting a possible threshold-like response in protein accumulation at the highest combined application intensity. At Carboj, the same trend was observed, with values increasing from 10.38% in S0 to 14.85% in S7. Fertilization increased protein content at Sparacia, from 11.83% to 13.73%, whereas no significant effect of fertilization was observed at Carboj.

The significant A × F interaction for yellow index at Sparacia showed that colour-related traits were influenced by the combination of microbial application and fertilization (**Figure 4**). In the unfertilized plots, most microbial treatments showed relatively homogeneous values, whereas S7 × F reached the highest value. This suggests that the most intensive microbial treatment, when combined with fertilization, did not compromise the yellow colour of the grain and may have supported a favourable chromatic profile.

**Figure 5:**
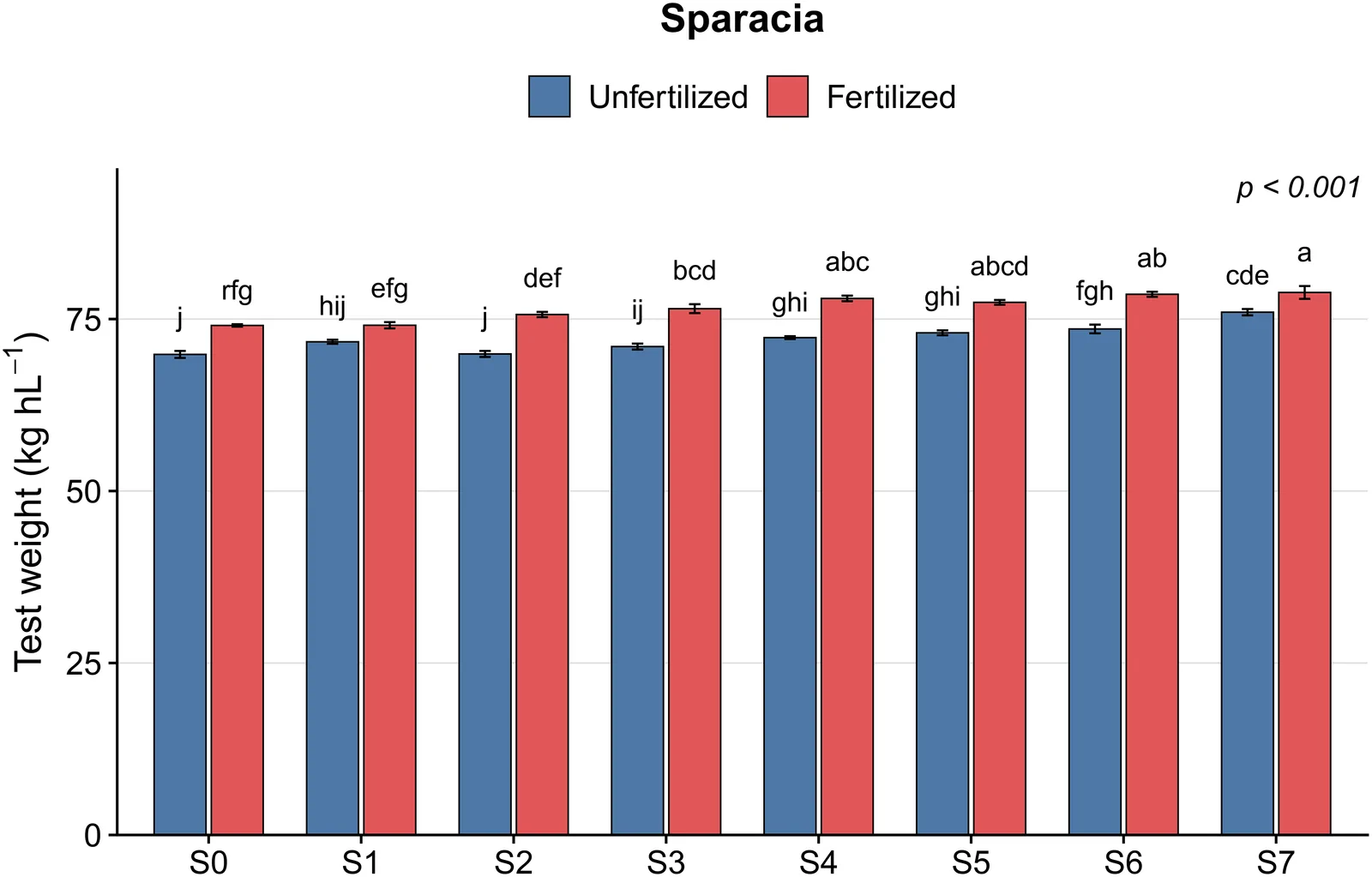
Influence of the application × fertilization interaction on test weight at Sparacia. For each data series, values with different letters are significantly different at p ≤ 0.05, according to Tukey’s HSD test. Error bars represent standard error. S0: soil spraying with water only; S1: seed coating at a dose of 100 g q-1 of seeds; S2: soil spraying after sowing at a dose of 1 kg ha-1 of microbial complex; S3: soil spraying after sowing at a dose of 2 kg ha-1 of microbial complex; S4: soil spraying after sowing at a dose of 3 kg ha-1 of microbial complex; S5: S1 + S2; S6: S1 + S3; S7: S1 + S4; F, fertilization; noF, unfertilized.

The significant A × F interaction observed for test weight at Sparacia showed that both microbial application and fertilization contributed to increasing grain density (**Figure 5**). In unfertilized plots, test weight increased progressively from S0 to S7, reaching 76.00 kg hL^-1^ in S7 × noF. Fertilization further increased test weight across treatments, with the maximum value recorded in S7 × F. Treatments S4, S5, and S6 under fertilization also showed high values, suggesting that combined microbial application and mineral fertilization improved grain physical quality in this site.

**Figure 6:**
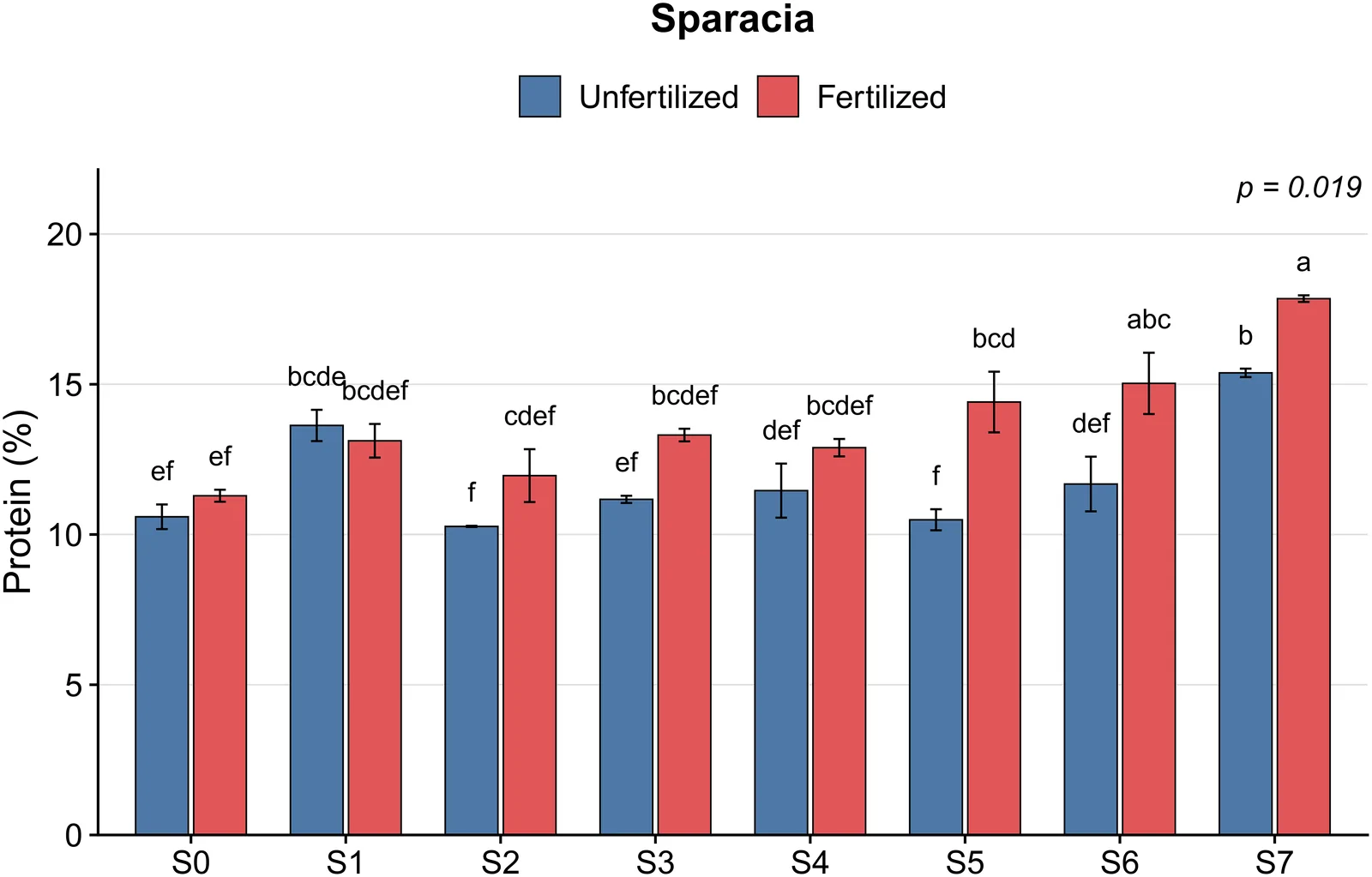
Influence of the application × fertilization interaction on grain protein content at Sparacia. For each data series, values with different letters are significantly different at p ≤ 0.05, according to Tukey’s HSD test. Error bars represent standard error. S0: soil spraying with water only; S1: seed coating at a dose of 100 g q-1 of seeds; S2: soil spraying after sowing at a dose of 1 kg ha-1 of microbial complex; S3: soil spraying after sowing at a dose of 2 kg ha-1 of microbial complex; S4: soil spraying after sowing at a dose of 3 kg ha-1 of microbial complex; S5: S1 + S2; S6: S1 + S3; S7: S1 + S4; F, fertilization; noF, unfertilized.

The A × F interaction for protein content at Sparacia showed a clear treatment-dependent response (**Figure 6**). In unfertilized plots, S1 and S7 produced the highest protein values, with S7 × noF reaching 15.38%. Under fertilization, the highest value was observed in S7 × F (17.85%), which was significantly higher than all other treatments. High protein values were also recorded in S5 × F and S6 × F, supporting the positive effect of combined seed + soil microbial applications on grain protein accumulation, particularly when associated with mineral fertilization.

### 3.3 Chemical composition and mineral content

Microbial application and mineral fertilization significantly affected grain chemical composition and mineral content at both sites (**Table 5**). For phosphorus, nitrates, calcium, and ash content, both main factors were significant in Sparacia and Carboj. The A × F interaction showed a more selective response: at Sparacia, it was significant for nitrates and ash only, whereas at Carboj it was significant for all mineral parameters.

**Table 5:** Analysis of variance (ANOVA) results for phosphorus (mg kg^-^¹), nitrates (mg kg^-^¹), calcium (mg kg^-^¹) and ash (%) in response to application (A), fertilization (F) and their interaction (A × F) at the two experimental sites (Sparacia and Carboj).

Source of Variation		df	Phosphorus [mg kg^-1^]	Nitrates [mg kg^-1^]	Calcium [mg kg^-1^]	Ash [%]
Application (A)	Sparacia	7	24.43 ***	125.74 ***	78.32 ***	29.39 ***
	Carboj		43.65 ***	520.89 ***	33.25 ***	30.34 ***
Fertilization (F)	Sparacia	1	10.06 **	127.16 ***	51.85 ***	43.26 ***
	Carboj		27.09 ***	1418.27 ***	37.82 ***	43.61 ***
A × F	Sparacia	7	0.72 ns	14.65 ***	2.15 ns	2.50 *
	Carboj		6.03 ***	47.36 ***	4.80 ***	2.41 *

Values are Fisher’s F statistics; degrees of freedom (df) are reported. Significance levels: ***p ≤ 0.001, **p ≤ 0.01, *p ≤ 0.05; ns, not significant.

Grain phosphorus concentration increased with microbial application in both sites (**Table 6**). At Sparacia, values ranged from 2638.25 mg kg^-1^ in S0 to 3561.75 mg kg^-1^ in S7, corresponding to an increase of approximately 35%. At Carboj, S7 also recorded the highest phosphorus concentration (3582.92 mg kg^-1^), closely followed by S6 (3495.08 mg kg^-1^). Mineral fertilization further increased grain phosphorus concentration, with fertilized plots averaging 3158.35 mg kg^-1^ at Sparacia and 3086.04 mg kg^-1^ at Carboj, compared with 3011.98 and 2874.44 mg kg^-1^ in the corresponding unfertilized plots.

**Table 6:** Effects of application and fertilization on grain chemical composition and mineral content: phosphorus (mg kg^-^¹), nitrates (mg kg^-^¹), calcium (mg kg^-^¹) and ash (%) at the two experimental sites (Sparacia and Carboj).

Treatments	Phosphorus [mg kg^-1^]		Nitrates [mg kg^-1^]		Calcium [mg kg^-1^]		Ash [%]	
	Sparacia	Carboj	Sparacia	Carboj	Sparacia	Carboj	Sparacia	Carboj
Application (A)								
S0	2638.25 ± 63.2 d	2592.67 ± 69.4 d	1940.25 ± 221 f	1418.00 ± 66.9 f	231.75 ± 6.12 e	238.00 ± 11.1 d	2.02 ± 0.05 d	1.77 ± 0.17 e
S1	3128.67 ± 61.9 bc	2991.58 ± 149 bc	2824.75 ± 57.2 bc	2117.00 ± 112 c	301.91 ± 9.17 b	311.58 ± 13.8 b	2.83 ± 0.13 bc	2.45 ± 0.04 bc
S2	2692.25 ± 87.9 d	2702.92 ± 59.0 d	2243.75 ± 73.9 e	1464.0 ± 46.7 f	242.33 ± 5.70 de	271.0 ± 8.32 c	2.07 ± 0.03 d	1.95 ± 0.14 de
S3	2927.83 ± 65.1 cd	2652.58 ± 96.9 d	2429.50 ± 46.6 d	1610.25 ± 95.4 e	260.16 ± 5.99 cd	308.08 ± 12.6 b	2.53 ± 0.11 c	2.05 ± 0.13 cd
S4	3076.58 ± 60.8 bc	2771.00 ± 78.1 cd	2754.0 ± 42.9 c	1704.25 ± 114 d	274.00 ± 5.63 c	322.75 ± 16.4 b	2.63 ± 0.09 c	2.25 ± 0.11 cd
S5	3293.83 ± 84.8 ab	3055.17 ± 79.4 b	2961.92 ± 57.3 ab	2122.25 ± 80.0 c	302.58 ± 7.81 b	273.58 ± 6.18 c	3.11 ± 0.19 ab	2.61 ± 0.12 ab
S6	3362.17 ± 77.4 ab	3495.08 ± 75.3 a	2990.0 ± 27.7 ab	2247.00 ± 106 b	320.16 ± 7.61 b	322.16 ± 8.42 b	3.15 ± 0.19 ab	2.79 ± 0.05 a
S7	3561.75 ± 58.9 a	3582.92 ± 94.8 a	3084.00 ± 56 a	2583.00 ± 230 a	344.83 ± 7.03 a	364.91 ± 6.16 a	3.30 ± 0.16 a	2.92 ± 0.04 a
p	0.000	0.000	0.000	0.000	0.000	0.000	0.000	0.000
Fertilization (F)								
no Fertilization (no F)	3011.98 ± 65.6 b	2874.44 ± 77.4 b	2508.79 ± 102 b	1663.0 ± 64.4 b	273.02 ± 6.69 b	286.70 ± 8.72 b	2.50 ± 0.07 b	2.17 ± 0.10 b
Fertilization (F)	3158.35 ± 73.0 a	3086.04 ± 87.4 a	2798.25 ± 63.9 a	2150.69 ± 102 a	296.02 ± 8.89 a	316.31 ± 8.62 a	2.91 ± 0.12 a	2.52 ± 0.06 a
p	0.003	0.000	0.000	0.000	0.000	0.000	0.000	0.000

Means and standard errors are reported. Within each column, values with different letters are significantly different at p ≤ 0.05, according to Tukey’s HSD test. S0: soil spraying with water only; S1: seed coating at a dose of 100 g q^-^¹ of seeds; S2: soil spraying after sowing at a dose of 1 kg ha^-^¹ of microbial complex; S3: soil spraying after sowing at a dose of 2 kg ha^-^¹ of microbial complex; S4: soil spraying after sowing at a dose of 3 kg ha^-^¹ of microbial complex; S5: S1 + S2; S6: S1 + S3; S7: S1 + S4.

Grain nitrate concentration also increased markedly with microbial application and fertilization. At Sparacia, values ranged from 1940.25 mg kg^-1^ in S0 to 3084.00 mg kg^-1^ in S7, while at Carboj they ranged from 1418.00 mg kg^-1^ in S0 to 2583.00 mg kg^-1^ in S7. Fertilization increased nitrate concentration in both sites, with a stronger absolute effect at Carboj. These results indicate that nitrogen-related grain composition responded strongly to the combined effect of microbial application and mineral fertilization, although the agronomic and technological meaning of nitrate accumulation should be discussed cautiously.

Calcium concentration followed a similar trend. At Sparacia, calcium increased from 231.75 mg kg^-1^ in S0 to 344.83 mg kg^-1^ in S7, while at Carboj it increased from 238.00 to 364.91 mg kg^-1^. Fertilization also increased calcium concentration at both sites, with mean values rising from 273.02 to 296.02 mg kg^-1^ at Sparacia and from 286.70 to 316.31 mg kg^-1^ at Carboj.

Ash content, used as an overall indicator of mineral accumulation in the grain, reflected the same general pattern. At Sparacia, ash content increased from 2.02% in S0 to 3.30% in S7, while at Carboj it increased from 1.77% to 2.92%. Fertilization significantly increased ash content in both sites. Overall, the chemical composition data show that the most intensive microbial applications, particularly S5, S6, and S7, consistently increased grain mineral accumulation compared with the untreated control.

**Figure 7:**
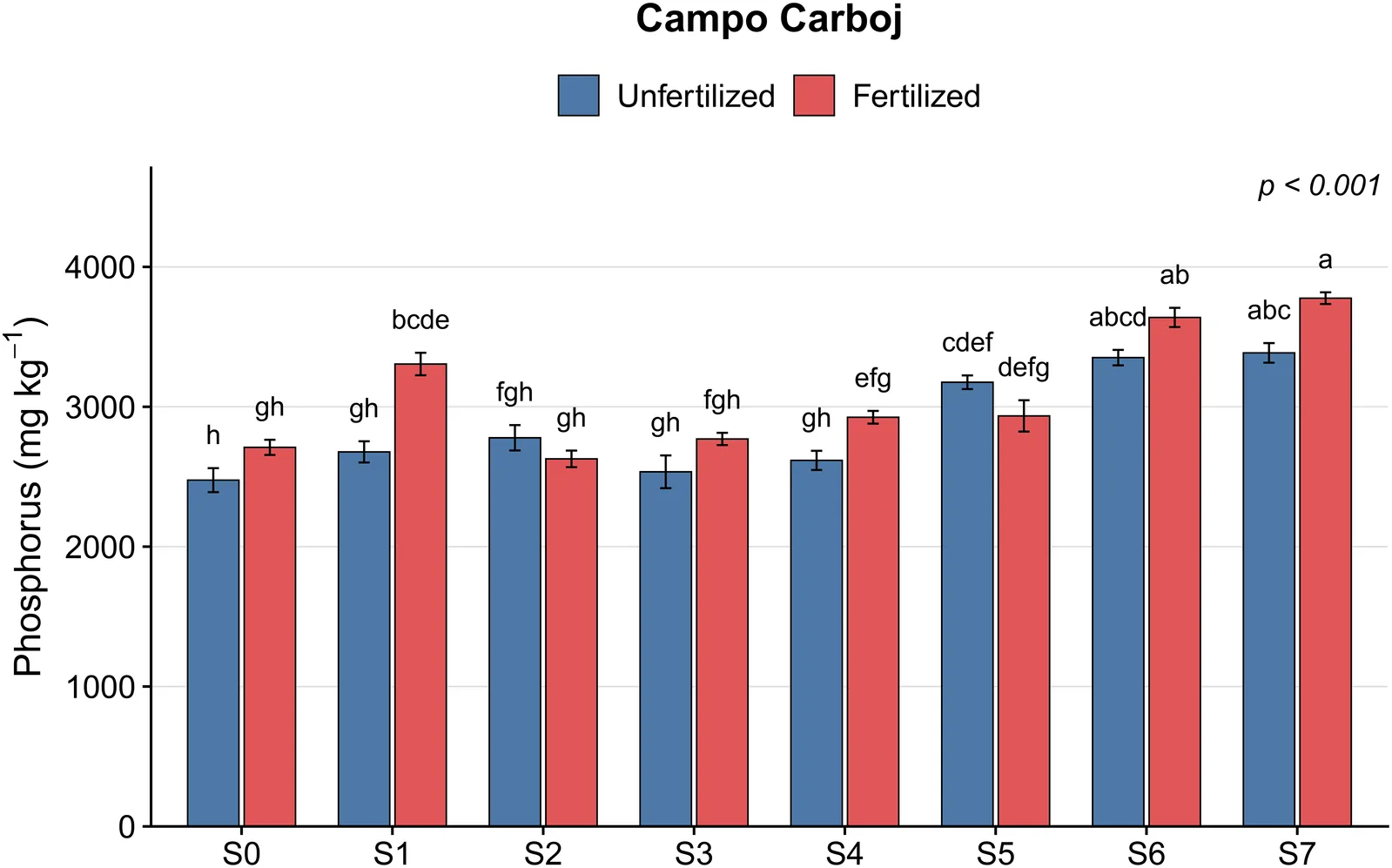
Influence of the application × fertilization interaction on grain phosphorus content at Campo Carboj. For each data series, values with different letters are significantly different at p ≤ 0.05, according to Tukey’s HSD test. Error bars represent standard error. S0: soil spraying with water only; S1: seed coating at a dose of 100 g q-1 of seeds; S2: soil spraying after sowing at a dose of 1 kg ha-1 of microbial complex; S3: soil spraying after sowing at a dose of 2 kg ha-1 of microbial complex; S4: soil spraying after sowing at a dose of 3 kg ha-1 of microbial complex; S5: S1 + S2; S6: S1 + S3; S7: S1 + S4; F, fertilization; noF, unfertilized.

At Carboj, where the A × F interaction for phosphorus was significant, grain P ranged from 2475.33 mg kg^-1^ in S0 × noF to 3776.50 mg kg^-1^ in S7 × F (**Figure 7**). S7 × noF also showed a strong increase compared with the unfertilized control, indicating that the highest microbial application increased grain P accumulation even in the absence of mineral fertilization. Among fertilized treatments, S6 × F and S7 × F formed the highest group, confirming the strong response of grain P concentration to combined microbial and mineral inputs.

**Figure 8:**
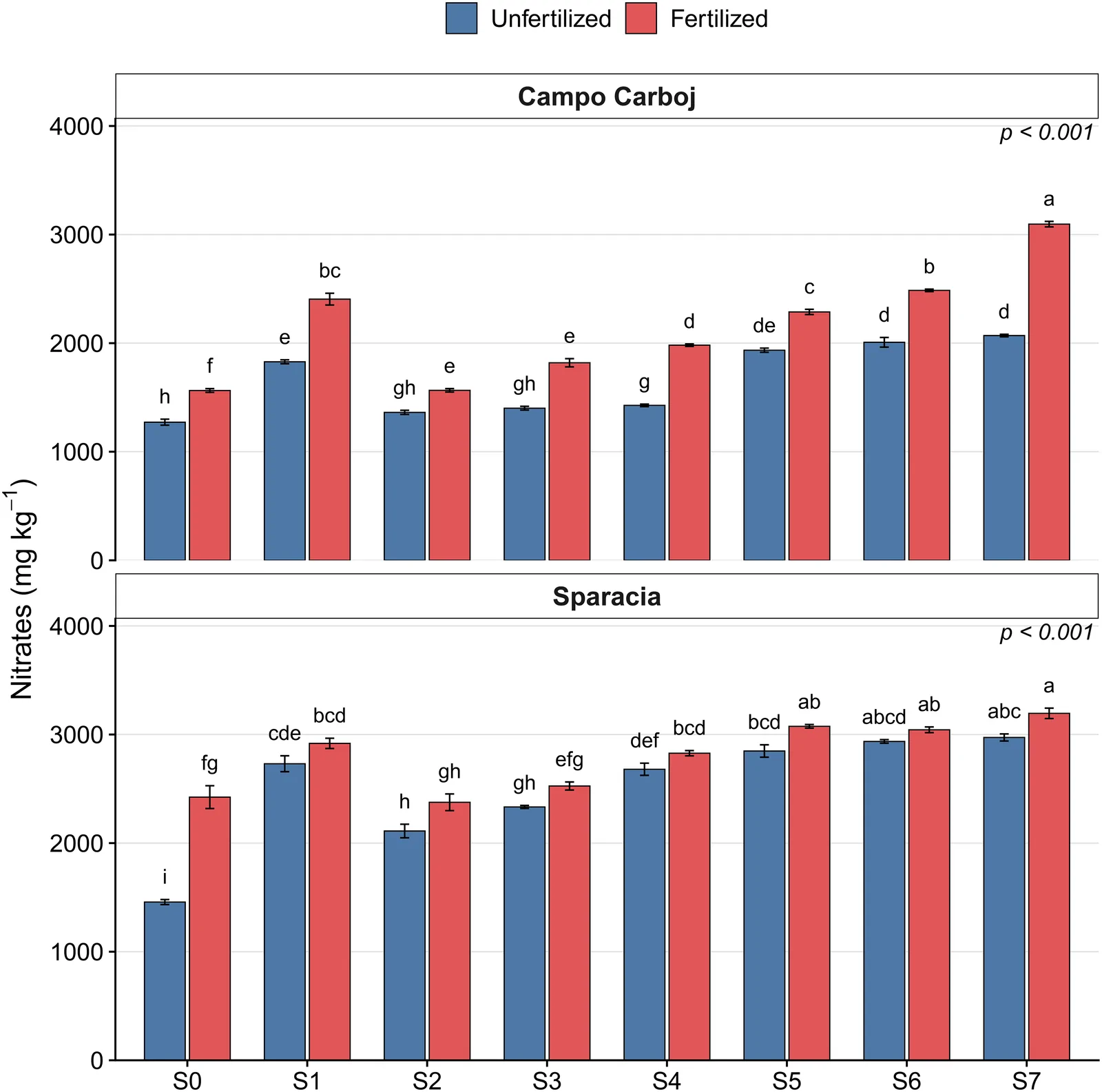
Influence of the application × fertilization interaction on grain nitrate content at the two Sicilian sites. Campo Carboj site; Sparacia site. For each data series, values with different letters are significantly different at p ≤ 0.05, according to Tukey’s HSD test. Error bars represent standard error. S0: soil spraying with water only; S1: seed coating at a dose of 100 g q-1 of seeds; S2: soil spraying after sowing at a dose of 1 kg ha-1 of microbial complex; S3: soil spraying after sowing at a dose of 2 kg ha-1 of microbial complex; S4: soil spraying after sowing at a dose of 3 kg ha-1 of microbial complex; S5: S1 + S2; S6: S1 + S3; S7: S1 + S4; F, fertilization; noF, unfertilized.

The A × F interaction for nitrates was significant in both environments (**Figure 8**). In both sites, S7 × F recorded the highest values, whereas the untreated and unfertilized controls showed the lowest nitrate concentrations. The response was particularly marked at Carboj, where fertilization strongly increased nitrate accumulation across microbial application levels.

**Figure 9:**
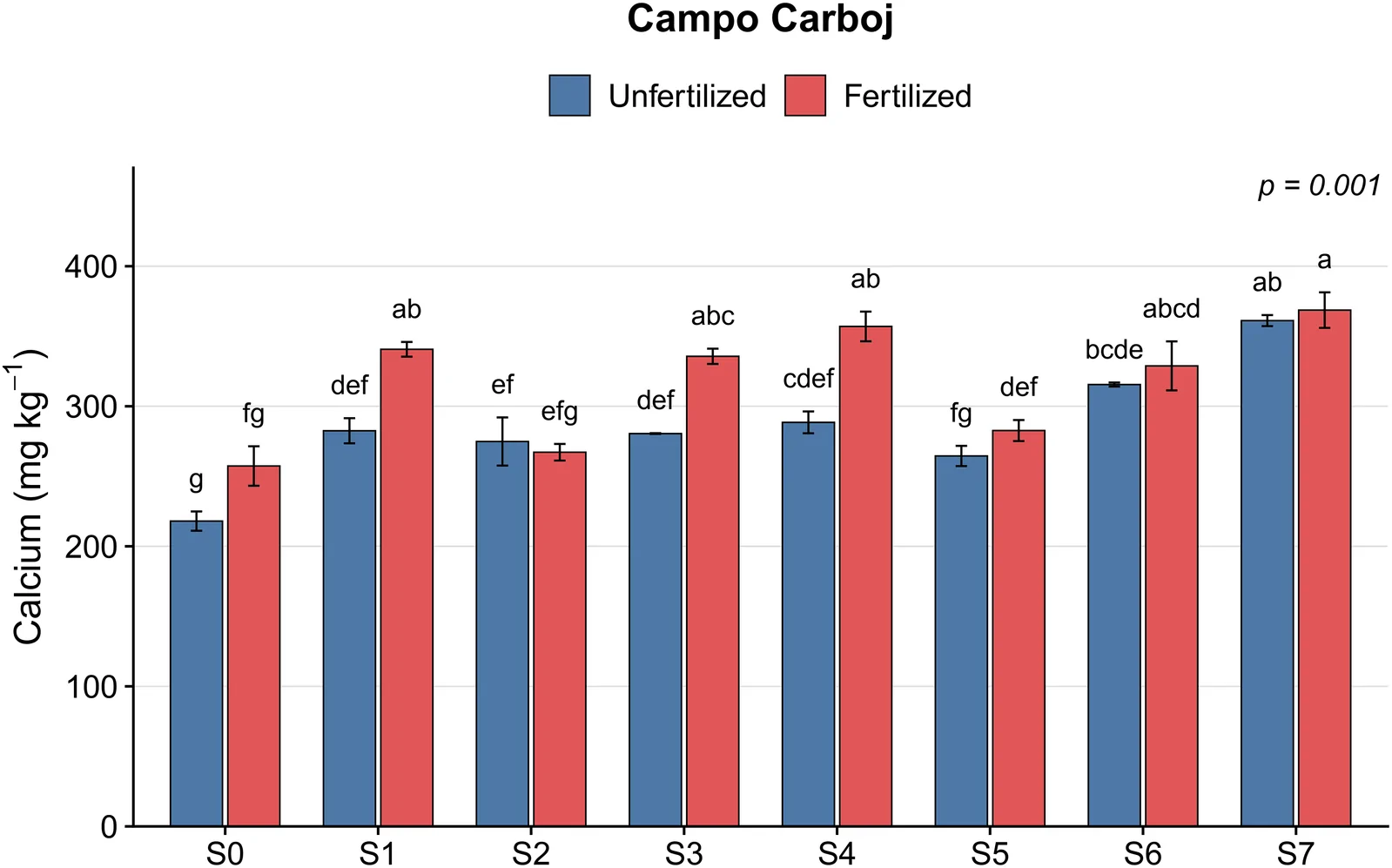
Influence of the application × fertilization interaction on grain calcium content at Campo Carboj. For each data series, values with different letters are significantly different at p ≤ 0.05, according to Tukey’s HSD test. Error bars represent standard error. S0: soil spraying with water only; S1: seed coating at a dose of 100 g q-1 of seeds; S2: soil spraying after sowing at a dose of 1 kg ha-1 of microbial complex; S3: soil spraying after sowing at a dose of 2 kg ha-1 of microbial complex; S4: soil spraying after sowing at a dose of 3 kg ha-1 of microbial complex; S5: S1 + S2; S6: S1 + S3; S7: S1 + S4; F, fertilization; noF, unfertilized.

At Carboj, the significant A × F interaction for calcium showed that S7 × F reached the highest value, although S7 × noF showed a statistically comparable response (**Figure 9**). This suggests that the highest microbial treatment strongly enhanced calcium accumulation even without mineral fertilization.

**Figure 10:**
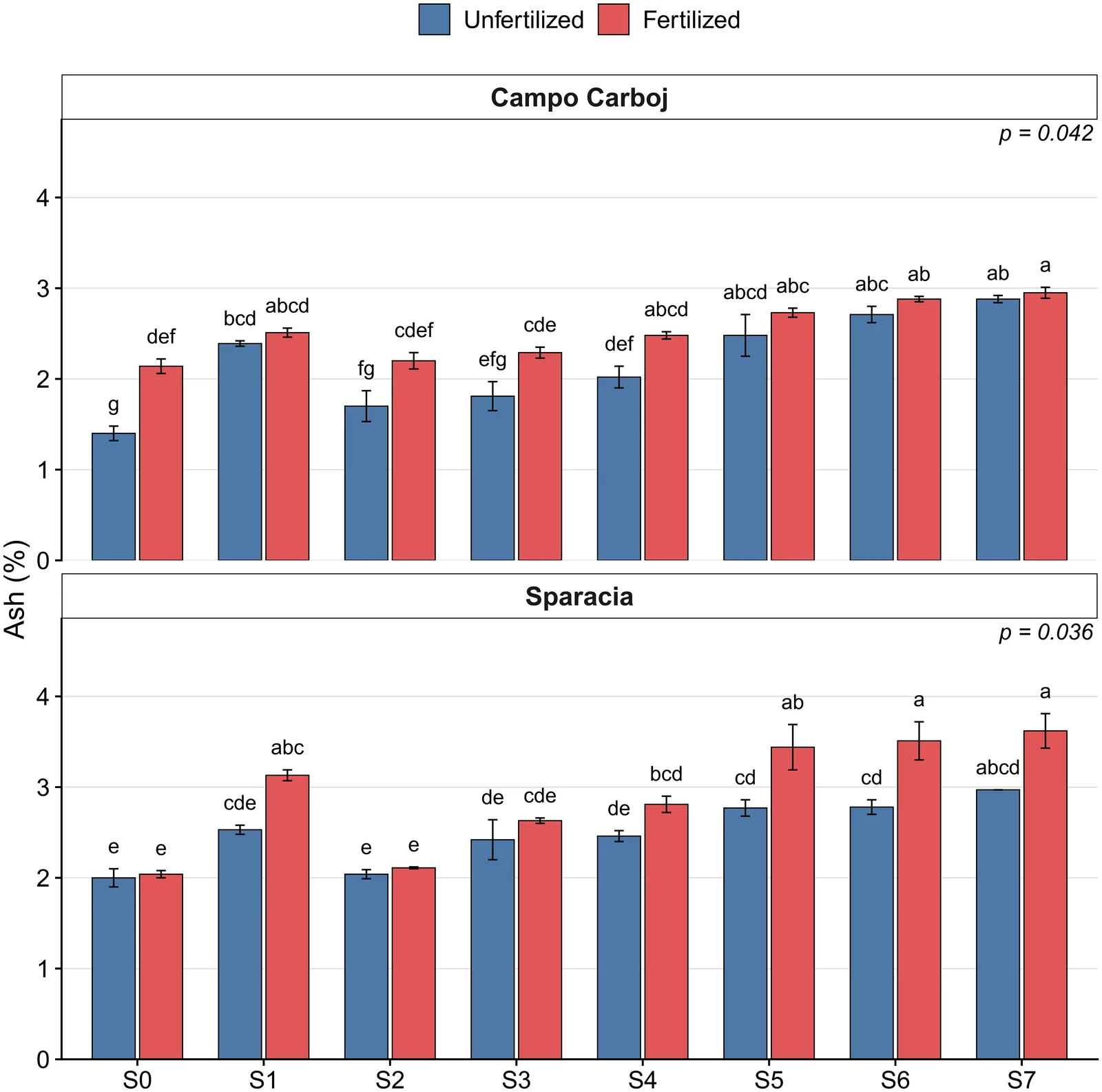
Influence of the application × fertilization interaction on grain ash content at the two sicilian sites. Campo Carboj site; Sparacia site. For each data series, values with different letters are significantly different at p ≤ 0.05, according to Tukey’s HSD test. Error bars represent standard error. S0: soil spraying with water only; S1: seed coating at a dose of 100 g q-1 of seeds; S2: soil spraying after sowing at a dose of 1 kg ha-1 of microbial complex; S3: soil spraying after sowing at a dose of 2 kg ha-1 of microbial complex; S4: soil spraying after sowing at a dose of 3 kg ha-1 of microbial complex; S5: S1 + S2; S6: S1 + S3; S7: S1 + S4; F, fertilization; noF, unfertilized.

For ash content, the A × F interaction was significant at both sites (**Figure 10**). The highest values were generally recorded under the combined microbial and fertilized treatments. At Sparacia, S5 × F, S6 × F, and S7 × F reached the highest ash contents, while at Carboj S7 × F showed the strongest increase relative to the untreated unfertilized control.

## 4 Discussion

The results of this study show that the application of the *Bacillus* consortium affected not only grain yield and aboveground biomass, but also grain filling, technological quality, and mineral composition. Overall, the response was strongly dependent on site, fertilization regime, and application strategy, confirming that the agronomic performance of microbial inoculants cannot be interpreted independently of the soil and environmental context (O'Callaghan et al., 2022). The most consistent improvements were observed with the combined seed + soil applications, particularly S6 and S7, while single-mode applications produced more variable and generally weaker responses. The magnitude of the response differed markedly between Sparacia and Campo Carboj, a pattern that was already evident in the physiological and soil nutrient data of Part I and that is further reinforced by the yield and quality results presented here.

The productivity data indicate that microbial application improved grain yield at both sites, although through partially different response patterns. At Sparacia, aboveground biomass increased by approximately 141% (from 32.13 q ha^-^¹ in S0 to 77.41 q ha^-^¹ in S7), whereas the increase in grain yield was limited to 33.0% increase (from 15.11 q ha^-^¹ in S0 to 20.09 q ha^-^¹ in S7). This suggests that, under the more restrictive conditions of this site, the microbial consortium strongly stimulated vegetative growth, but only part of this additional biomass was translated into grain production. The heavy clay texture at Sparacia (62% clay, pH 7.89) imposes mechanical resistance to root expansion, restricts soil aeration, and promotes strong chemical adsorption of phosphorus onto colloidal and calcium carbonate surfaces (Brownrigg et al., 2022; von Wandruszka, 2006). These edaphicconstraints restrict root exploration and slow down nutrient diffusion, particularly during the critical grain-filling phase under typical Mediterranean terminal drought. Under these highly limiting conditions, the *Bacillus* consortium exerted its greatest relative biostimulatory benefit. By solubilizing P from immobilized fractions and releasing organic acids, the inoculant alleviated both nutrient starvation and water-deficit stress, thereby partially compensating for the physical limitations of the root system. According to broader PGPR literature, members of the genus *Bacillus* can further support plant resilience under stress through the synthesis of phytohormones (such as auxins and cytokinins) and ACC deaminase activity (Cataldi et al., 2020; Khourchi et al., 2022; Benmrid et al., 2024; Kudoyarova et al., 2019). Although these specific hormonal and enzymatic traits were not directly assayed in the present study, such literature-backed pathways provide plausible physiological hypotheses for the observed field biostimulation. Furthermore, site-specific differences in soil water dynamics and microbial ecology likely played a pivotal role in shaping the crop response. In the heavy clay soil of Sparacia, water availability during the late reproductive stages declines rapidly due to high soil water tension, creating a severe physiological bottleneck for nitrogen and phosphorus translocation to the developing grain. The observed stimulation of biomass (+141%) and protein accumulation at Sparacia suggests that the inoculated bacilli may have enhanced the crop’s physiological resilience to terminal drought, possibly by maintaining cell turgor or modifying root hydraulic conductivity through biological pathways. From a biological perspective, the native soil microbial communities at the two sites likely exerted different levels of “biological buffering” against the introduced strains. In the highly fertile, sandy-loam soil of Campo Carboj (59% sand), a diverse and highly active native microbial community may have presented a competitive barrier to the establishment of the introduced *Bacillus* consortium. This would explain why, at Campo Carboj, only the most intensive and multimodal delivery strategies (such as S7, combining seed coating and high-rate soil spraying) were able to overcome this biological resistance and induce significant agronomic benefits, such as closing the yield gap in unfertilized plots. Conversely, at Sparacia, where heavy clay soils often exhibit lower microbial biomass and restricted biological activity due to poor aeration, the introduced consortium likely found a more accessible ecological niche, allowing even lower-intensity treatments (S4–S6) to colonize the rhizosphere effectively and promote substantial vegetative and qualitative improvements. Nevertheless, the disproportion between biomass increase and grain yield increase also indicates that microbial stimulation of vegetative growth does not necessarily result in a proportional improvement in final yield, especially in Mediterranean environments where terminal water and heat stress frequently impair grain filling and source–sink efficiency during the reproductive phase (Cossani and Reynolds, 2012; Tortorici et al., 2025).

At Campo Carboj, the response pattern was different. The absolute grain yield was higher than at Sparacia with the intensive S7 treatment leading to a 35.1% increase in grain yield (rising from 21.71 q ha^-^¹ in the control to 29.33 q ha^-^¹). When evaluating the main factors, both the Bacillus consortium and nitrogen fertilization exerted significant independent effects on yield and quality parameters across sites, whereas their interaction was non-significant for several key variables. This lack of a significant statistical interaction indicates that the beneficial biostimulatory effects of the microbial inoculants occurred consistently across the different nitrogen fertilization levels, rather than being strictly dependent on a specific nitrogen dose. Consequently, the PGPR consortium and mineral nitrogen inputs functioned as parallel, additive drivers of crop performance, each independently optimizing distinct steps of resource acquisition and translocation to the grain. This independent but complementary action is consistent with recent evidence showing that *Bacillus*-based biofertilizers can optimize nutrient use efficiency and partially offset mineral inputs in wheat production under specific soil and management conditions (Aslam et al., 2024; Długosz and Piotrowska-Długosz, 2023). Similar responses have been reported for phosphate-solubilizing *Bacillus* strains and microbial consortia, whose benefits tend to become more evident when nutrient availability limits crop performance, but whose effectiveness remains strongly dependent on strain performance, soil properties, and crop management (Wang et al., 2022; Afzal et al., 2023; Elhaissoufi et al., 2025).

The thousand grain weight results further support the hypothesis that microbial inoculation improved grain filling, particularly when combined with mineral fertilization. The response was more pronounced at Campo Carboj, where S6 × F and S7 × F formed the highest statistical group contributing to an overall TGW increase of 22.8% in S7 compared to the control (from 40.84 g to 50.17 g). This suggests that, in a less restrictive soil environment, the combined effect of microbial application and mineral fertilization enhanced assimilate availability and translocation during the source–sink dynamics of the filling period, resulting in heavier and denser grains. At Sparacia, TGW also increased, but within a narrower range (a moderate 8.2% increase, from 35.65 g in S0 to 38.56 g in S7), probably because the same edaphic constraints that limited yield also constrained the full expression of grain weight potential. In this sense, TGW represents an important intermediate trait linking the physiological improvements observed during the crop cycle — as documented in Part I — with the final productivity response at harvest. The positive effect of microbial inoculation on TGW is consistent with previous studies showing that phosphate-solubilizing bacteria can improve wheat growth, nutrient acquisition, and yield components (Afzal et al., 2023; Erdemci, 2021; Sonkurt and Çiğ, 2019). It is noteworthy that these effects extend beyond P nutrition alone: Erdemci (2021) demonstrated that seed coating with *Bacillus*-based consortia improved TGW and test weight in durum wheat through multifaceted mechanisms involving enhanced nutrient uptake efficiency and phytohormone modulation. While biochemical hormone profiles were not quantified in our trial, the observed gains in grain filling similarly point toward a comprehensive physiological biostimulation rather than an isolated effect on P availability. Similar improvements in yield components following PSB inoculation have also been reported in other cereal crops, further supporting the generality of these mechanisms across environments (Beltran-Medina et al., 2023).

The technological quality traits also showed a clear site-dependent response. Dry gluten was mainly influenced by mineral fertilization, especially at Campo Carboj, where microbial application alone did not produce significant differences. This result is expected, since gluten accumulation is strongly linked to nitrogen availability and grain protein formation. By contrast, protein content was significantly affected by microbial application at both sites, with S7 consistently showing the highest values (16.61% at Sparacia and 14.85% at Campo Carboj). This is one of the most agronomically relevant results of the study, because the highest microbial application increased protein content without an evident yield trade-off. In Mediterranean cereal systems, protein concentration and yield are frequently inversely related: under terminal heat and water stress, grain filling is shortened, reducing starch accumulation while concentrating nitrogen-containing compounds in a smaller grain mass (Tortorici et al., 2025). This “concentration effect” can artificially inflate protein percentage without reflecting genuine improvement in nitrogen acquisition or nutritional quality. In the present study, however, S7 and the combined treatments achieved simultaneous improvements in grain yield, TGW, and protein content, suggesting that the consortium supported a genuine enhancement of nitrogen-related grain composition rather than a stress-induced concentration effect. The most intensive combined applications may have improved the overall nutritional status of the crop through enhanced P availability, improved root functioning, and better acquisition or remobilization of nitrogen — a response consistent with evidence showing that microbial inoculants can promote nutrient transporter expression and improve N and P uptake efficiency in durum wheat (Saia et al., 2015). In this regard, Dal Cortivo et al. (2020) reported that seed-applied biofertilizers containing *Bacillus megaterium* modified rhizosphere biochemistry and early root development in *Triticum aestivum*, supporting the view that microbial application can alter the nutritional trajectory of the crop from early establishment onwards; the present study, conducted on durum wheat under Mediterranean field conditions with a multi-modal inoculation strategy, appears to have extended these early-stage effects into measurable grain quality improvements at harvest. These mechanisms should nonetheless be interpreted cautiously, as root architecture, microbial colonization dynamics, N uptake fluxes, and N remobilization were not directly measured in this study.

Test weight responded positively to both microbial application and fertilization, with the A × F interaction significant at Sparacia but not at Campo Carboj. At Sparacia, where soil P immobilization and physical constraints were more pronounced, the combination of microbial application and mineral fertilization produced a synergistic improvement in grain density that neither factor alone could fully achieve, reflecting better grain filling under more limiting conditions. The absence of a significant A × F interaction at Campo Carboj likely reflects the more homogeneous baseline soil fertility of that site, where mineral fertilization alone was sufficient to support a strong grain density response, leaving less room for additional microbial modulation. From a technological perspective, test weight is directly associated with milling performance and semolina extraction efficiency (Wang and Fu, 2020), and the increases observed in the most intensive microbial treatments — especially when combined with mineral fertilization — suggest a potential benefit not only in terms of field productivity but also in terms of grain commercial quality.

The yellow index showed a more variable response. At Sparacia, the significant A × F interaction suggests that colour-related traits were influenced by the combination of microbial application and fertilization, whereas at Campo Carboj the response was mainly driven by fertilization alone. Colour is an important commercial quality parameter in durum wheat, as it is closely associated with carotenoid pigment concentration in the semolina and strongly influences pasta appearance and consumer acceptance. When grain yield increases, carotenoid concentration can be diluted in a larger grain mass, leading to a reduction in yellow index even when total pigment production remains unchanged. Ibarra-Villarreal et al. (2023), applying a *Bacillus*-based consortium on durum wheat, reported a slight reduction in yellow index associated with yield increases, attributing this to a dilution effect of carotenoid pigments in larger grain. Notably, in the present dataset, this dilution effect did not occur in S7 × F at Sparacia, which achieved both the highest yield-related values and the highest yellow index (26.94%). This outcome suggests that the most intensive combined application, when associated with mineral fertilization, was able to support carotenoid accumulation proportional to grain size throughout filling, possibly because the improved nutritional status of the crop - driven by enhanced P availability and root activity - sustained the metabolic pathways involved in pigment biosynthesis during the late reproductive stages. This result carries direct practical relevance, since semolina colour is one of the primary quality criteria used by the pasta industry, and maintaining a high yellow index while increasing yield represents a commercially valuable combination that is rarely achieved under conventional agronomic management alone.

The chemical and mineral composition data provide an important link with Part I of this study. Grain phosphorus concentration increased with microbial application at both sites, especially under S6 and S7. At Sparacia, P concentration rose by approximately 35% (reaching 3561.75 mg kg^-^¹ in S7), while at Campo Carboj, it peaked at 3582.92 mg kg^-^¹ under S7. This result is coherent with the increase in soil available P_2_O_5_ documented in the companion paper and supports the hypothesis that the *Bacillus* consortium improved the entire soil-plant P pathway, from P mobilization in the soil to P accumulation in the grain. Although the specific mechanisms were not directly measured here, this response is consistent with the broader evidence that phosphate-solubilizing microorganisms promote soil P transformation and plant P acquisition through organic acid release, phosphatase activity, and rhizosphere-mediated changes in P availability (Pang et al., 2024). Similar effects have been reported for *Bacillus*-based inoculants increasing P acquisition and crop productivity under reduced or limiting P availability (Khourchi et al., 2022; Wang et al., 2022; De Oliveira-Paiva et al., 2024). Importantly, fertilization further increased grain P concentration, indicating that microbial application and mineral nutrient supply were not mutually exclusive but often acted in a complementary way.

Calcium and ash content followed a similar pattern, with ash content rising from 2.02% to 3.30% at Sparacia, and from 1.77% to 2.92% at Campo Carboj in the S7 treatments. The increase in ash content suggests a broader improvement in mineral accumulation rather than an isolated effect on phosphorus, consistent with findings showing that microbial inoculation can improve the uptake of multiple mineral elements in wheat (Moreno-Lora et al., 2023; Ibnyasser et al., 2024). In this context, a key quantitative outcomewas observed at Campo Carboj, where the unfertilized intensive treatment (S7 × noF) achieved a grain calcium concentration statistically comparable to the fertilized counterpart (S7 × F). This suggests that the *Bacillus* consortium might have contributed to maximizing calcium bioavailability independently of mineral inputs. A plausible, literature-backed explanation for this trend is the exudation of organic acids and chelating agents capable of solubilizing calcium carbonates typical of Mediterranean soils. Nevertheless, since root traits and rhizosphere chemical changes were not directly measured in the present study, it is impossible to definitively untangle whether this grain mineral enrichment was driven by specific microbial solubilization pathways or by a generalized improvement in overall root absorption capacity. Given that calcium acts as a crucial second messenger regulating stomatal conductance and membrane stability under thermal and drought stress, this potential microbial-driven biofortification could represent a significant physiological asset for durum wheat coping with terminal stress in semi-arid environments. In this regard, it is important to clarify that calcium (Ca) biofortification differs fundamentally from the more conventional iron (Fe) and zinc (Zn) biofortification. While Fe and Zn are micro-elements whose accumulation in the grain relies heavily on late-stage phloem remobilization from vegetative tissues assisted by chelators, Ca is a plant macro-element that is strictly phloem-immobile. Consequently, grain Ca enrichment cannot occur via leaf remobilization; instead, it depends exclusively on continuous vascular xylem delivery driven by root pressure and transpiration during early grain filling. PGPR, such as the strains tested here, facilitate this specific process by solubilizing soil-bound minerals via organic acid excretion and optimizing root absorption capacity, thereby increasing root-to-shoot xylem flux rather than modulating intracellular phloem transport. This could reflect enhanced root activity, improved nutrient uptake, or greater translocation of minerals to the grain. Nevertheless, the mechanisms underlying these changes remain indirect in the present study. Without direct field measurements of root traits, rhizosphere chemistry, microbial persistence, or real-time nutrient uptake dynamics during the crop cycle, we cannot definitively distinguishwhether the observed grain mineral enrichment was driven mainly by enhanced P solubilization, improved root growth, changes in rhizosphere pH, altered enzyme activity, or a combination of these processes. Consequently, these proposed physiological mechanisms are treated strictly as plausible hypotheses to be verified in future mechanistic studies. Furthermore, caution is required when comparing our open-field results with the greenhouse or pot studies frequently cited in the literature. Controlled environment experiments offer valuable mechanistic insights under optimized moisture, temperature, and light regimes with minimal microbial competition. However, extrapolating such findings directly to field conditions is inherently limited. Open-field agroecosystems expose inoculated PGPR to severe environmental fluctuations, erratic rainfall, soil physical/chemical heterogeneity, and fierce competitive interactions with the resident soil microbiome. These abiotic and biotic stressors can significantly influence bacterial survival, root colonization efficiency, and overall metabolic performance compared to controlled conditions. Therefore, while greenhouse literature supports the biological plausibility of our findings, field-scale validation under diverse pedoclimatic conditions remains essential to accurately evaluate the operational consistency of microbial biofertilizers.

The nitrate data require a particularly cautious interpretation. Nitrate concentration increased with microbial application and fertilization, especially in the highest-intensity treatments, a pattern that mirrors the observed increase in protein content but requires an explicitly nuanced interpretation. Crucially, grain nitrate accumulation must not be automatically interpreted as an improvement in technological or nutritional grain quality, as high unassimilated nitrate levels do not contribute to pasta-making properties and can be sub-optimal from a food safety perspective.These data are best interpreted as a biochemical indicator of altered nitrogen-related grain composition and shifts in internal nitrogen metabolism. Physiologically, this accumulation likely reflects a temporary kinetic imbalance between accelerated root nitrogen uptake and the plant’s downstream assimilation capacity during the grain filling phase. It is highly plausible that the intensive microbial treatments stimulated a rapid flux of nitrate (NO_3_^-^) into the developing sink that temporarily outpaced the reduction capacity of the nitrate reductase enzyme system, leading to incomplete nitrate reduction and subsequent storage as free nitrate rather than organic nitrogen. Agronomically, the marked increase in grain nitrate concentration under the most intensive microbial treatments points toward a potential modulation of nitrogen dynamics within the rhizosphere by the inoculated strains. Strains within the *Bacillus* genus have been reported in literature to stimulate organic matter mineralization and interact positively with native nitrifying prokaryotes, which could conceptually increase the flux of available nitrate (NO_3_^-^) for root uptake (Etesami and Adl, 2020). The high accumulation in the grain could tentatively suggest that the hypothesized microbial-driven nitrogen uptake during the post-anthesis phase temporarily outpaced the reduction capacity of the plant’s nitrate reductase enzyme, leading to the storage of unassimilated nitrates in the sink. However, as rhizosphere nitrogen fluxes and enzyme activities were not directly monitored in this trial, these interactions remain speculative and require direct biochemical validation. This suggests a potential shift in the overall nutritional trajectory of the crop, altering the baseline of nitrogen availability even under restricted chemical fertilization.

Taken together, the results indicate that the *Bacillus* consortium did not simply produce a uniform increase across all traits. Instead, it modulated plant performance in a trait-specific and site-specific manner. At Sparacia, where soil constraints were stronger, the microbial treatments had a pronounced effect on biomass, protein, test weight, and mineral accumulation, suggesting that inoculation improved crop functionality under limiting conditions. At Campo Carboj, where baseline conditions supported higher absolute productivity, the main agronomic relevance was the near-elimination of the yield gap between fertilized and unfertilized plots at the highest inoculation level. This distinction highlights two different, and complementary, modes of action: mitigation of soil-related constraints in the more limiting environment, and partial compensation of mineral fertilization in the more productive one.

The better performance of the combined seed + soil applications confirms that delivery strategy is a key determinant of inoculant effectiveness in field conditions (O'Callaghan et al., 2022). It could be hypothesized that in fertile, less-constraining soils such as Campo Carboj, simple seed coating should suffice, given the inherent physiological resilience of *Bacillus* strains and their ability to form resistant endospores under nutritional or water stress. However, our field results demonstrate that booster soil applications (e.g., S7) provided superior and more consistent agronomic outcomes compared to seed coating alone. This phenomenon can be explained through rhizosphere population dynamics: while spore formation ensures physical survival, active plant growth promotion (such as organic acid exudation for mineral solubilization) relies on metabolically active vegetative cells. In biologically active, fertile soils, introduced strains originating solely from the seed coat face fierce competition from the native soil microbiome and undergo spatial dilution as the root architecture expands into deeper layers. Booster soil applications overcome this “biotic resistance” by continuously replenishing active vegetative populations in the topsoil, thereby sustaining high cell density and metabolic activity during the critical stages of crop growth. The combination of both approaches may therefore provide a more stable and persistent microbial effect than either application method alone, a hypothesis consistent with the general view that field performance of microbial inoculants depends not only on strain traits, but also on formulation, delivery method, soil conditions, and crop phenology (O’Callaghan et al., 2022).

It is also worth contextualizing these findings beyond the Mediterranean cereal production model. In Mediterranean agroecosystems, rainfed durum wheat relies heavily on stored soil moisture and is systematically subject to terminal water and heat stress during grain filling, making root biostimulation and P/Ca solubilization critical for alleviating physiological bottlenecks (Tortorici et al., 2025). By contrast, major global wheat-producing systems outside the Mediterranean basin - such as the continental steppe regions of Ukraine and Russia or the US Great Plains - operate under distinct pedoclimatic dynamics. In the deep, organic matter-rich soils of Eastern Europe (Ukraine and Russia), moisture is often less limiting during early grain filling, but crops face harsh winter freezing and severe temperature swings; in such continental environments, *Bacillus*-based biostimulants are primarily leveraged to enhance early cold tolerance, root crown establishment, and mobilization of organic soil nutrient pools (Koryagin et al., 2020; Kucher et al., 2025). Similarly, in the US Great Plains, where wheat production spans extensive dryland and irrigated systems under highly variable continental rainfall, microbial inoculants are predominantly deployed to maximize nitrogen use efficiency (NUE) and offset fertilizer costs at large operational scales (Di Benedetto et al., 2017; Chaudhary et al., 2022). Acknowledging these regional differences highlights that while the biostimulatory mechanisms of *Bacillus* consortia (e.g., P solubilization, root system architecture modulation) are universally valuable, their primary agronomic benefit shifts from drought-stress mitigation in Mediterranean climates to nutrient-use optimization and abiotic cold resilience in major continental producing zones.

Overall, the present study supports the agronomic potential of *Bacillus*-based inoculation as a complementary tool for improving durum wheat productivity and grain quality under Mediterranean field conditions. The most intensive combined applications, particularly S7, consistently improved several productive, technological, and mineral traits. However, the results should be interpreted as evidence of partial and context-dependent compensation of mineral fertilization, not as proof of generalized fertilizer replacement. Future studies should test intermediate fertilizer reduction levels, include multi-year validation, and measure root traits, microbial persistence, rhizosphere P dynamics, and plant N/P uptake to clarify the mechanisms underlying the observed improvements and define the conditions under which *Bacillus*-based inoculation can reliably contribute to more sustainable durum wheat production in the Mediterranean.

## 5 Conclusions

This study demonstrates that the agronomic value of the tested *Bacillus* consortium extends beyond the improvements in soil phosphorus availability and plant physiological performance reported in the companion paper (Part I), resulting in measurable benefits for grain productivity, technological quality, and nutritional composition at harvest. By integrating the findings of both studies, the results provide a comprehensive understanding of how microbial inoculation can influence the entire soil–plant continuum, from rhizosphere nutrient mobilization to final grain performance.

Among the tested delivery strategies, the combined seed + soil applications, particularly S7 and, to a lesser extent, S6, consistently produced the greatest agronomic benefits. These treatments simultaneously improved grain yield, thousand-grain weight, protein concentration, test weight, and grain phosphorus and calcium accumulation, indicating that the microbial consortium enhanced both productivity and grain quality without evidence of the typical yield–protein trade-off. The superior performance of the combined applications further demonstrates that inoculation strategy is a critical determinant of field efficacy and should be considered alongside strain selection when developing microbial-based fertilization programs.

The response of the consortium was strongly site-dependent. Under the more restrictive soil conditions of Sparacia, microbial inoculation primarily alleviated edaphic constraints by improving biomass production, grain quality, and mineral accumulation. In contrast, under the more productive conditions of Campo Carboj, the greatest practical outcome was the substantial reduction of the productivity gap between fertilized and unfertilized plots under the most intensive inoculation strategy. These contrasting responses firmly indicate that the principal agronomic role of the consortium is strictly non-substitutive, acting not as a universal replacement of mineral fertilizers, but as a dedicated biological tool for the enhancement of nutrient use efficiency according to site-specific environmental and soil conditions.

Overall, the combined evidence from Parts I and II supports the use of this multi-strain *Bacillus* consortium exclusively as a complementary and supportive component of sustainable fertilization strategies for Mediterranean durum wheat. The consortium is not intended to replace mineral fertilization; instead, it improved the efficiency with which both endogenous and exogenous soil nutrients were converted into grain yield and quality, highlighting its potential to contribute to more resource-efficient cropping systems.

Future research should validate these findings across multiple years, environments, soil types, and durum wheat cultivars while evaluating progressive fertilizer-reduction scenarios to define the agronomic and economic limits of mineral fertilizer replacement. In addition, from a biotechnological perspective, integrating soil and rhizosphere microbiological analyses will be essential. While the strains were co-formulated based on *in vitro* biochemical compatibility, the relative colonization percentage and persistence of each individual *Bacillus* strain in the complex wheat rhizosphere were not quantified in this field study. Therefore, future research utilizing strain-specific quantitative PCR (qPCR) or high-resolution metagenomic sequencing will be critical to track the real-time colonization dynamics and relative proportions of individual strains, as well as to determine how inoculation reshapes native microbial communities throughout the crop cycle. Coupling these analyses with direct measurements of root development, nutrient uptake dynamics, and rhizosphere phosphorus transformations would provide the mechanistic evidence needed to explain the observed field responses and to optimize microbial inoculation strategies for sustainable Mediterranean wheat production.

## Data Availability

The raw data supporting the conclusions of this article will be made available by the authors, without undue reservation.
